# Shotgun Proteomics of Tomato Fruits: Evaluation, Optimization and Validation of Sample Preparation Methods and Mass Spectrometric Parameters

**DOI:** 10.3389/fpls.2016.00969

**Published:** 2016-06-29

**Authors:** Himabindu V. Kilambi, Kalyani Manda, Hemalatha Sanivarapu, Vineet K. Maurya, Rameshwar Sharma, Yellamaraju Sreelakshmi

**Affiliations:** Repository of Tomato Genomics Resources, Department of Plant Sciences, School of Life Sciences, University of HyderabadHyderabad, India

**Keywords:** tomato fruit, shotgun proteomics, proteome coverage, sample preparation, protein fractionation

## Abstract

An optimized protocol was developed for shotgun proteomics of tomato fruit, which is a recalcitrant tissue due to a high percentage of sugars and secondary metabolites. A number of protein extraction and fractionation techniques were examined for optimal protein extraction from tomato fruits followed by peptide separation on nanoLCMS. Of all evaluated extraction agents, buffer saturated phenol was the most efficient. In-gel digestion [SDS-PAGE followed by separation on LCMS (GeLCMS)] of phenol-extracted sample yielded a maximal number of proteins. For in-solution digested samples, fractionation by strong anion exchange chromatography (SAX) also gave similar high proteome coverage. For shotgun proteomic profiling, optimization of mass spectrometry parameters such as automatic gain control targets (5E+05 for MS, 1E+04 for MS/MS); ion injection times (500 ms for MS, 100 ms for MS/MS); resolution of 30,000; signal threshold of 500; top *N*-value of 20 and fragmentation by collision-induced dissociation yielded the highest number of proteins. Validation of the above protocol in two tomato cultivars demonstrated its reproducibility, consistency, and robustness with a CV of < 10%. The protocol facilitated the detection of five-fold higher number of proteins compared to published reports in tomato fruits. The protocol outlined would be useful for high-throughput proteome analysis from tomato fruits and can be applied to other recalcitrant tissues.

## Introduction

Tomato is a good model for fleshy fruit ripening due to the availability of a high quality genome sequence, mutant collections, well characterized wild relatives, ease of transformation, etc. It is extensively used for deciphering the molecular basis for fruit ripening at transcriptome and metabolome levels (Gapper et al., 2014; Pesaresi et al., 2014). Proteome is an essential link that connects transcriptome and metabolome (Gapper et al., 2014). As proteins functionally represent the genome, proteome profiling identifies the regulatory components mediating diverse pathways, as well as the proteins that can serve as markers for improving nutritional quality, flavor, disease resistance/tolerance, shelf life, etc.

To capture the complete repertoire of the proteins present in plant tissue, a robust extraction protocol coupled with efficient peptide fractionation and identification by MS is essential. The major challenge underlying proteome profiling is the sheer number and the wide dynamic range of proteins constituting the protein complement. Moreover, plant tissues pose additional challenges owing to high level of proteases, and presence of primary and secondary metabolites that interfere with protein extraction (Saravanan and Rose, 2004). Though several protocols are available for protein extraction (Isaacson et al., 2006; Wang et al., 2008) from fruit tissues like apple, banana (Carpentier et al., 2005; Amoako-Andoh et al., 2014), grape (Vincent et al., 2006), tomato (Saravanan and Rose, 2004) for 2DE-based proteomics, very few exist for shotgun proteomics.

Currently, shotgun proteomics is the most preferred method for proteome profiling (McCormack et al., 1997). For profiling complex mixtures of proteins, 2D-LC in combination with MS/MS, MudPIT (Multidimensional Protein Identification Technology) is used (Washburn et al., 2001; Motoyama and Yates, 2008). Although several labeling techniques are available for proteome profiling, their inherent limitations including high costs and incomplete labeling have made label-free quantification a more feasible choice for researchers (Patel et al., 2009).

Given the complexity of the samples and the wide dynamic range of protein abundances, optimization of LC and MS parameters are utmost for optimal detection of proteins. For shotgun proteomics, data dependent acquisition is the most widely used mode (Stahl et al., 1996) and includes a number of MS parameters for data collection. In tomato fruits, shotgun proteomics for obtaining proteome profiles was used in few studies wherein, GeLCMS using purified chromoplast proteins (Barsan et al., 2012; Wang et al., 2013) or SCX (strong cation exchange chromatography) using proteins from fruit pericarp (Osorio et al., 2011) were employed prior to separation on nanoLCMS. However, no rationale was given for selecting any of these methods. Considering the difficulties involved in protein extraction, the effect of various LC conditions (Peterson et al., 2009; Xu et al., 2009) and MS parameters (Wong et al., 2009; Zhang et al., 2009; Andrews et al., 2011; Kalli and Hess, 2012) on proteome coverage, we optimized sample preparation protocols and MS parameters for proteome profiling of tomato fruits. We assessed various reported methods, modified the protein extraction, fractionation protocols and evaluated their effect on final proteome profiles. Based on the results, we incorporated the MS parameters that had a significant impact on proteome coverage into a data dependent method and validated it across two tomato cultivars. Here we report an optimized protocol for shotgun proteomics of tomato fruits.

## Materials and methods

### Plant material

Tomato (*Solanum lycopersicum*) cultivars, Arka Vikas (AV) and Ailsa Craig (AC) were grown in a greenhouse at 25 ± 2°C. Flowers were tagged at anthesis and fruits were collected at the red ripe stage. Each fruit was cut into four halves, deseeded, frozen in liquid nitrogen and stored at −80°C until use. For all experiments, AV fruit tissue was used whereas, AC and AV fruit tissue were used for validation experiments.

### Protein extraction

#### With buffer saturated phenol

Proteins were extracted following the protocol described by Kilambi et al. (2013). Briefly, 1 g of frozen tissue was homogenized and suspended in 7 mL of extraction buffer containing 0.7 M Sucrose, 0.1 M KCl, 0.5 M Tris, pH 7.5, 50 mM EDTA, 50 mM dithiothreitol (DTT), 1 mM phenyl methyl sulfonyl fluoride (PMSF), and 25 μL of protease inhibitor cocktail (Sigma-Aldrich). To this, an equal volume of Tris-saturated phenol was added, and the sample was mixed by shaking at 4°C for 30 min. The mixture was centrifuged at 20,000 g for 30 min at 4°C. The upper phenolic phase was collected and re-extracted twice as described above. The protein in the phenolic phase was precipitated at −80°C by adding 5 volumes of 0.1 M ammonium acetate containing 50 mM DTT. The protein was pelleted by centrifugation at 26,200 g for 30 min at 4°C. The protein pellet was washed twice with methanol containing 10 mM DTT followed by a wash with acetone containing 10 mM DTT. The pellet was stored at −80°C until further use.

#### With trichloroacetic acid (TCA)

Proteins were extracted using the protocol described by Ippoushi et al. (2015) with slight modifications. Briefly, 100 mg of frozen tissue was homogenized and suspended in 1 mL of 10% (w/v) TCA in 80% (v/v) acetone containing 2% (w/v) DTT. The mixture was incubated overnight at −20°C. Thereafter mixture was centrifuged at 14,000 g at 4°C for 30 min and the supernatant was discarded. The pellet was rigorously washed with 10 mL of 80% (v/v) acetone, and centrifuged at 14,000 g for 10 min at 4°C. The resulting supernatant was discarded and the acetone wash of pellet was repeated as described above. Protein pellet obtained after two acetone washes was stored at −80°C until further use.

#### With SDS

**A**. Proteins were extracted with 1 mL of SDS buffer^a^ (0.5 M Tris pH 6.8, 50% (v/v) glycerol, 10% (w/v SDS), 0.2 M DTT) using an earlier protocol (Mora et al., 2013). Briefly, 1 g of homogenized tissue was suspended in 1 mL of SDS buffer^a^, boiled at 95°C for 30 min. The resulting mixture was centrifuged at 5000 g for 5 min. The supernatant was precipitated with 80% (v/v) acetone by incubating at −80°C for 3 h. The protein pellet obtained after centrifugation at 20,000 g for 20 min was washed with 100% acetone and stored at −80°C until further use.

**B**. Proteins were extracted with SDS buffer^b^ (4% (w/v) SDS, 100 mM Tris-HCl, pH 7.6, 0.1 M DTT) following the protocol described in Wisniewski et al. (2009). Briefly, 0.7 g of homogenized tissue was suspended in 7 mL of SDS buffer^b^ and boiled at 95°C for 30 min. The resulting mixture was centrifuged at 5000 g for 5 min and the supernatant was precipitated with 80% (v/v) acetone by incubating at −80°C for 3 h. The protein pellet obtained after centrifugation at 20,000 g for 20 min was washed with 100% (v/v) acetone and solubilized in 50 mM ammonium bicarbonate (ABC). The protein solution was dialyzed for 16–18 h against 20 mM Tris-HCl pH 8.0. The protein solution was used further for digestion with trypsin and peptide fractionation.

**C**. Proteins were extracted with SDS buffer^c^ (20 mM Tris-HCl pH 8.8, 2% (w/v) SDS) following the protocol described in Tanca et al. (2013). About 1 g of homogenized tissue was suspended in 1 mL of SDS buffer^c^ (20 mM Tris-HCl pH 8.8, 2% (w/v) SDS) boiled at 95°C for 30 min. The resulting mixture was centrifuged at 20,000 g for 20 min and the supernatant was diluted to a final concentration of 0.2% (w/v) SDS and precipitated with 80% (v/v) acetone by incubating at −80°C for 3 h. Protein pellet obtained after centrifugation at 20,000 g for 20 min was washed with 100% (v/v) acetone and stored at −80°C until further use.

#### With FASP (filter aided sample preparation)

Hundred milligram homogenized fruit tissue was solubilized in 1 mL of SDS buffer^b^, sonicated for 5 min and proteins were extracted by FASP protocol described in Wisniewski et al. (2009). Sample was centrifuged at 16,000 g for 5 min. The supernatant was transferred to filter unit (Millipore, YM30) and 200 μL of UA (8 M urea in 0.1 M Tris-HCl pH 8.5) was added and centrifuged at 14,000 g for 15 min. This step was repeated twice and the flow through was discarded. Then 100 μL of 50 mM iodoacetamide (IAA) was added and mixed at 600 rpm in thermomixer for 1 min at room temperature followed by incubation without mixing for 20 min. The filter units were then centrifuged at 14,000 g for 10 min followed by addition of 100 μL of UA. This was subjected for centrifugation at 14,000 g for 15 min. This step was repeated twice. Then 100 μL of 50 mM ABC was added and centrifuged at 14,000 g for 10 min. This step was repeated twice. Trypsin (Promega) was added to the filter in the ratio of 1: 100 (enzyme: protein) and mixed at 600 rpm on a thermomixer for 1 min. The filter units were then incubated in a wet chamber at 37°C for 16 h. After incubation, the filter units were transferred to new collection tubes and centrifuged at 14,000 g for 10 min. Peptides were eluted using 50 μL of 0.5 M sodium chloride and centrifuged at 14,000 g for 10 min. The eluate was acidified with 0.1% (v/v) formic acid, desalted using C18 spin columns (Pierce) and dried under vacuum. These samples were stored at −80°C until further use.

#### With FASP after phenol extraction

Proteins were extracted using phenol extraction protocol as described above and precipitated with ammonium acetate. This protein pellet was dissolved in UA (8M urea in 0.1 M Tris-HCl pH 8.5) and processed as per FASP protocol discussed above.

### Protein estimation

For all the discussed protocols, protein estimation was done using amido black method (Goldring and Ravaioli, 1996). The amount of protein considered for further downstream processing is 100 μg unless stated otherwise.

### Peptide fractionation

After obtaining pellets using extraction protocols described in the previous section, proteins were digested with trypsin at specific conditions using DTT, IAA as the common reagents. The concentrations of these components and the processing conditions varied according to the fractionation method employed and are described below.

### In-gel digestion-SDS-PAGE (GeLCMS)

Proteins (100 μg) obtained after phenol and TCA extraction were dissolved in 2D lysis buffer and then separated on SDS-PAGE according to Laemmli (1970). After destaining, the gel was cut into 36 slices, and six slices were pooled in a single fraction (total 6 fractions were obtained). The proteins were reduced with 10 mM DTT, alkylated with 55 mM IAA and subjected to trypsin digestion (1: 25, enzyme: protein) for 16 h at 37°C. Peptides from each fraction were separately extracted by addition of 60% (v/v) acetonitrile (ACN) containing 0.1% (v/v) formic acid and sonicated in ice for 30 min. This step was repeated thrice, and peptides obtained from each extraction step were pooled. The pooled peptides for each fraction were then concentrated using speed vacuum concentrator (Thermo Scientific), desalted using C18 spin columns and then subjected to LCMS analysis.

### In-solution digestion

**A**. Phenol-extracted proteins (100 μg) were dissolved in 2D lysis buffer (7 M urea, 2 M thiourea, 4% (w/v) CHAPS), reduced with 10 mM DTT, alkylated with 40 mM IAA. Urea concentration in the solution was reduced to 2 M by the addition of 50 mM ABC. This solution was then subjected to trypsin digestion (added in the ratio of 1: 50, enzyme: protein) at 37°C for 16 h. Peptides were concentrated, desalted prior to LCMS analysis.

**B**. Phenol-extracted proteins (100 μg) were dissolved in 10% (w/v) SDS buffer^a^, boiled at 95°C for 30 min. Samples were reduced with 10 mM DTT, alkylated with 40 mM IAA. SDS buffer concentration in the solution was reduced to 0.2% (w/v) SDS by the addition of 50 mM ABC. The solution was then subjected to trypsin digestion (added in 1: 50, enzyme: protein) at 37°C for 16 h. Peptides were concentrated, desalted and then subjected to LCMS analysis.

**C**. Phenol-extracted proteins (100 μg) were dissolved in 6 M Guanidium hydrochloride buffer and boiled at 95°C for 30 min (Yeats et al., 2010). Samples were reduced with 10 mM DTT, alkylated with 40 mM IAA. Guanidium hydrochloride concentration in the solution was reduced to 0.6 M by the addition of 50 mM ABC and subjected to trypsin digestion (added in 1:50, enzyme: protein) at 37°C for 16 h. Peptides obtained were concentrated, desalted and then subjected to LCMS analysis.

**D**. TCA-extracted proteins (100 μg) were digested as per the protocol described in Ippoushi et al. (2015). Extracted peptides were concentrated, desalted prior to LCMS analysis.

**E**. Proteins (100 μg) obtained after extraction with SDS buffer^a^ were solubilized in 1X Invitosol (Life Technologies)/0.1% (v/v) Rapigest (Waters) in 50 mM ABC. The resulting solution was vortexed for 5 min, reduced with 10 mM DTT, incubated at 60°C for 30 min (until the pellet was completely dissolved). After the samples were cooled to room temperature, 25 mM IAA was added and incubated in dark for 30 min. Trypsin was added to the samples in the ratio of 1:20 (enzyme: protein) and subjected to digestion at 37°C for 16 h. Formic acid (0.1% v/v) was added to the samples to stop digestion. Samples were centrifuged, supernatant was collected and peptides in the supernatant were concentrated, desalted and then subjected to LCMS analysis.

**F**. Proteins (100 μg) obtained after extraction with SDS buffer^b^ were digested after dialysis for 16–18 h. Trypsin was added to the sample in the ratio of 1:40 (enzyme: protein) and subjected to digestion at 37°C for 16 h. Formic acid (0.1% v/v) was added to the samples to stop digestion. Samples were centrifuged, peptides in the supernatant were concentrated, desalted and subjected to LCMS analysis.

**G**. Proteins (100 μg) obtained after extraction with SDS buffer^c^ were solubilized in 8 M Urea and incubated at room temperature for 30 min. The sample was then diluted with milliQ water to reduce the final urea concentration to 2 M, and subjected to digestion at 37°C for 16 h with trypsin. In the method where precipitation was not adopted, the concentration of SDS was reduced to 0.2% (w/v) by desalting. Protein samples were then reduced with 10 mM DTT by incubating at 56°C for 30 min. Samples were cooled to the room temperature, 25 mM IAA was added and incubated in dark for 30 min. Trypsin was added to the sample (1:20, enzyme: protein) and subjected to digestion at 37°C for 16 h. Formic acid (0.1% v/v) was added to the samples to stop digestion. Samples were centrifuged, peptides in the supernatant were concentrated, desalted and subjected to LCMS analysis.

**H**. Phenol-extracted proteins (100 μg) were dissolved in UA and processed as per FASP protocol discussed previously. Peptides obtained after digestion were desalted, dried and subjected to LCMS analysis.

**I**. Basic pH reverse phase liquid chromatography (bRPLC): bRPLC was carried out using peptides obtained after trypsin digestion of phenol extracted protein (500 μg and 1 mg) as per the protocol described in Renuse et al. (2014). The tryptic peptides were reconstituted in solvent A (10 mM trimethyl ammonium bicarbonate, pH 9.5) and fractionated using Accela UHPLC system (Thermo Scientific) on XBridge C18 column (250 × 4.6 mm, 5 μm, 200 A°, Waters Corporation, USA). The peptides were separated using a 10–40% linear gradient of solvent B (10 mM trimethyl ammonium bicarbonate, 90% (v/v) acetonitrile, pH 9.5) for 42 min at a flow rate of 1 mL/min. The fractions were collected in 42 individual tubes containing 10 μl of 20% (v/v) FA. Peptides were dried and then reconstituted in 60% (v/v) ACN and 0.2% (v/v) FA, concatenated into 20 fractions, desalted, dried and subjected to LCMS analysis.

### Separation based on charge

#### Strong anion exchange (SAX)

Phenol extracted proteins (100 μg) were reduced with 10 mM DTT followed by alkylation with 40 mM IAA. The concentration of urea was reduced to 2 M using 50 mM ABC and then subjected to trypsin digestion for 16 h at 37°C (1: 50, enzyme: protein). The obtained tryptic peptides were desalted, dried and reconstituted in Tris buffer (20 mM Tris-HCl, pH 8.0) and then loaded onto SAX column (2 mL of Q-Sepharose (GE Healthcare) packed in 5 mL syringe). Fractionation was done using step gradients of 0.1, 0.25, and 0.5 M NaCl, with 5 column volumes of each salt gradient. Each fraction was dialyzed for 16 h in Tris buffer (20 mM Tris-HCl pH 8.0) and thereafter desalted prior to LCMS analysis. Similar steps were carried out for peptides obtained from FASP protocol after phenolic extraction of proteins for FASP-SAX and also for peptides obtained from TCA extracted protein.

#### Strong cation exchange (SCX)

SCX was carried out using the protocol described by Renuse et al. (2014). Briefly, phenol extracted protein (500 μg) was digested with trypsin, and the peptides were dissolved in 1 mL of solvent A (10 mM KH_2_PO_4_, 20% v/v ACN, pH 2.8). The peptides were then separated on PolySulfoethyl A column (150 × 2.1 mm, 5 μm, 200 A°, PolyLC Inc., USA) using a linear gradient of 0–100% solvent B (10 mM KH_2_PO_4_, 350 mM KCl, 20% (v/v) ACN, pH 2.8) connected to HPLC system (Shimadzu) at a flow rate of 0.2 mL/min for 52 min. A total of 52 fractions were collected, completely dried, and reconstituted in 40 μl of 60% (v/v) ACN and 0.2% (v/v) FA. These fractions were pooled based on their peak profiles into 10 fractions, desalted, dried and injected into LCMS for further analysis.

#### Peptide IEF (PEP-IEF)

Peptide IEF was carried out based on the protocol developed by Atanassov and Urlaub (2013). Peptides obtained from phenol extraction (250 and 500 μg), phenol-FASP protein extraction (100 μg), and TCA extraction (100 μg) were dissolved in 8 M Urea and 0.2% (v/v) IPG (immobilized pH gradient) buffer (GE Healthcare). This peptide solution was applied to 4–7 pH/3-10 pH IPG strips and passively rehydrated overnight at 25°C followed by separation on IPGphor (GE Healthcare) at maximum of 50 μA per strip using the following conditions: 150 V for 1 h, 250 V for 1 h, 500 V for 30 min, 1750 Vh at a gradient of 500–3000 V, then 27,750 V h at 8000 V at 20°C. After peptide IEF, IPG strips were cleaned by immersing them in n-hexane for 10 s followed by slicing the gel into 12 pieces (1 cm each). Peptides were then extracted by incubating the gel slices for 30 min each with 1% (v/v) FA; 50% (v/v) ACN, 1% (v/v) FA; and 99% (v/v) ACN, 1% (v/v) FA. Extracted peptides were desalted, dried and subjected to LCMS analysis.

### LCMS analysis and optimization of MS parameters

Separation of peptides was carried out on Easy nanoLC-II coupled with LTQ Velos Pro mass spectrometer (Thermo Scientific). Tryptic peptides (350 ng) were loaded onto a trap column (Integrafrit 100 μm × 2.5 cm, 5 μm, C18 New Objective, USA) and eluted on a Biobasic C18 picofrit column (75 μm × 10 cm, 5 μm, New Objective, USA) using solvent A (95/5-water/ACN with 0.1% (v/v) formic acid) and solvent B (95/5-ACN/water with 0.1% (v/v) formic acid) at a flow rate of 350 nl/min. Different gradients were employed for samples obtained from various extraction protocols based on the complexity of the sample, fractionation technique employed, and the number of fractions collected and the details of gradients are listed in Table S1. Column eluate was connected to nanospray ionization source operated at a voltage of 1.7 kV and a capillary temperature of 260°C. Mass spectra were obtained through data dependent acquisition in positive mode. MS scans were performed in Orbitrap at a resolution of 60,000 within a scan range of 350–2000. Data dependent MS/MS scans were performed in ion trap using CID as the fragmentation technique. The activation energy of 35% and activation time of 10 ms was used for fragmentation. Dynamic exclusion of 30 s was enabled with a repeat count of 1 and a lock mass of 445.120030 was used for mass accuracy. An isolation window of 2 *m/z* was applied. Automatic gain control targets of 500 ms and 500,000 ions for FTMS and 100 ms and 10,000 ions for MS/MS were applied respectively.

Fraction 1 obtained from GeLCMS of phenol extracted samples (In-gel digested samples) was used for evaluation and optimization of MS parameters using an 118 min gradient for elution of peptides as given in Table S1. Conditions for loading tryptic peptides, solvents used for separation and ion source parameters employed are the same as described above. Data dependent parameters that were used in evaluation and optimization are listed in Table S2.

### Validation of protein preparation, fractionation and MS parameters

To check the consistency of the sample extraction/solubilization/fractionation conditions and the evaluated MS parameters, proteome profiling of ripe tomato fruits from two different cultivars—AV, an Indian cultivar grown predominantly in South India, and AC, a popular European cultivar was carried out. Phenol extraction followed by in-gel digestion (GeLCMS for all the cultivars) was employed for sample preparation in three biological replicates. The MS parameters that gave good results were incorporated into a data dependent method and the created instrument method having all the optimized parameters was used for validation.

Conditions for loading tryptic peptides, solvents used for separation and ion source parameters applied are the same as described in LCMS analysis section. MS scans were performed in Orbitrap at a resolution of 60,000 within a scan range of 350–2000. Data dependent MS/MS scans were performed in ion trap using CID as the fragmentation mode. Monoisotopic precursor selection was enabled and the signal threshold value was set to 500. Dynamic exclusion of 30 s was enabled with a repeat count of 1 and a lock mass of 445.120030 was used for mass accuracy. An isolation window of 2 *m/z* was applied. Activation energy of 35 eV and activation time of 10 ms was used for fragmentation. Fragmentation was carried out for top 20 peaks obtained from each survey scan. Automatic gain control targets of 500 ms and 500,000 ions for MS and 100 ms and 10,000 ions for MS/MS (Combination I) were applied respectively.

### Data analysis

Data analysis was done using Proteome Discoverer (version 1.4, Thermo Scientific). *S. lycopersicum* iTAG2.3 proteome sequence (ftp://ftp.solgenomics.net/tomato_genome/annotation/ITAG2.3_release/ITAG2.3_proteins.fasta, downloaded on February 5, 2013, 26,705 sequences and 9,322,189 residues) was used as the database against which the searches were done. Peptide mass tolerance and fragment mass tolerance were set to 5 ppm and 0.8 Da respectively. Sequest was used as the search engine with the following search parameters- trypsin as the protease, a maximum of two missed cleavages were allowed, carbamidomethylation of cysteine and oxidation of methionine were selected as fixed and variable modifications respectively. Peptides were filtered for high confidence and these were used for assigning protein IDs. Percolator tool was used to assess peptide confidence; peptides with *q* ≤ 0.05 were selected (false discovery rate (FDR) of 1%), which was estimated based on the number of decoy hits. Proteins that passed the criteria of high confidence with XCorr threshold greater than 2.0 and a minimum number of two matched peptides were considered. Information regarding the number of MS and MS/MS scans, fill times were obtained from RawMeat version 2.1. The mass spectrometry proteomics data have been deposited to the ProteomeXchange Consortium (Vizcaíno et al., 2014) via the PRIDE partner repository with the dataset identifier PXD003920.

### Gene ontology analysis

Proteins identified in this study were annotated based on their molecular function, biological process and cellular component with Gene Ontology (GO) annotation using ProteinCenter (version 1) in Proteome Discoverer 1.4.

### Label-free quantitation

The raw data obtained from the mass spectrometer were analyzed using Scaffold software (version 4.4.8, Proteome Software) using the parameters and the database as mentioned above in data analysis. Peptides were filtered for high confidence (95% protein and peptide probabilities, assigned through Protein Prophet algorithm; Nesvizhskii et al., 2003) and these were used for assigning protein IDs. An FDR cut off of 1% which was estimated through a local FDR database was used to filter the peptides on the basis of number of decoy hits. Label-free quantification was performed by spectral counting method in Scaffold. The protein abundances were calculated through normalized spectral abundance factor (NSAF), which divides the weighted spectrum count for each protein by the length of the same protein and the results are then normalized across all samples (Zybailov et al., 2006). Fisher's exact test was applied on the proteins with a match of minimum two peptides and FDR of 0.1% to identify the statistically significant proteins based on their *P*-values. The proteins which have passed the criteria of minimum two peptides, two-fold up- or downregulation and a *P*-Value of less than 0.05 were considered as significantly different between AC and AV cultivars.

### Carotenoid profiling

Carotenoid content in the red ripe fruits of AV and AC was determined using a previously published protocol (Gupta et al., 2015).

## Results and discussion

Ideally, the protein extraction protocol should reproducibly capture most of the protein repertoire from a given biological matrix with minimal protein degradation and minimal contamination of extraneous components. The presence of high levels of soluble sugars, cell wall polysaccharides, organic acids and the aromatic ring containing secondary metabolites in tomato fruits interferes and leads to suboptimal protein extraction (Saravanan and Rose, 2004). Few studies (Saravanan and Rose, 2004; Wang et al., 2008; Wu et al., 2014) have critically reviewed phenol and TCA/acetone for protein extraction and precipitation in a variety of plant tissues for proteome profiling using 2DE. However, their applicability to shotgun proteomics remains to be established. In this study, we examined the suitability of sample preparation protocols on final proteome coverage using shotgun proteomics approaches. Additionally, efforts were made to simplify the protocol by altering the components, using filter aids and consequential differences in protein identification were examined.

### Protein extraction with buffer-saturated phenol followed by precipitation is optimal for sample preparation from tomato fruit

**Figure 1:**
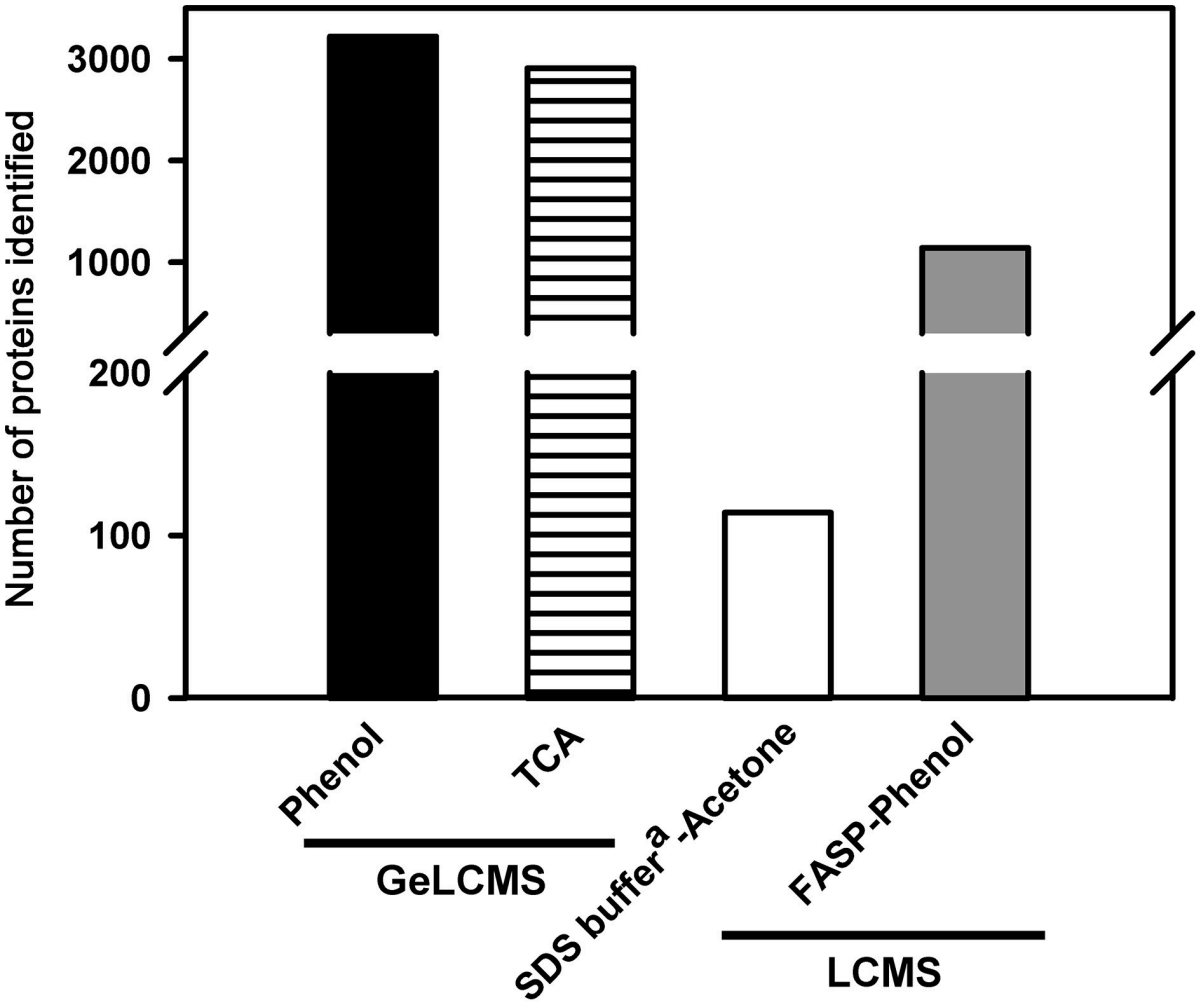
**Effect of different extraction and precipitation reagents on protein identification**. Only the proteins identified with ≥2 peptide matches are shown. The details of the buffer composition, reagent concentrations, and conditions are described in methods.

Among the extraction methods tested, protein extraction with buffer-saturated phenol followed by precipitation with ammonium acetate in methanol and subsequent fractionation (either by in-gel/in solution-IEC) yielded highest number of proteins (3220) compared to proteins obtained with TCA/acetone (2907) (Figure 1). Though both phenol and TCA/acetone extraction show well resolved protein profiles on 2DE (Saravanan and Rose, 2004), our results revealed that phenol is better suited for extracting protein from tomato fruits for shotgun approaches. The efficacy of phenol likely resulted from the efficient partitioning of interfering substances into the aqueous phase while proteins were retained in phenol (Isaacson et al., 2006). In contrast, TCA reportedly precipitates polysaccharides along with proteins (Wu et al., 2014). Replacing phenol with SDS buffer^a^ (Figure 1), SDS buffer^b^ or SDS buffer^c^ (Table 1) for protein extraction resulted in total loss of proteins. Precipitation with chilled acetone after extraction with SDS buffer^a^ was grossly inadequate, yielding only 114 proteins (Figure 1). Above results indicated that precipitation of proteins after extraction in an ideal reagent is a crucial step, as in addition to efficient extraction of proteins, the reagent should also effectively eliminate the common contaminants.

**Table 1:** **Effect of solubilizing agents on protein identification**.

**Protocol**	**Protein extraction method**	**Buffer used for solubilization of protein pellet**	**Number of proteins identified**
In-solution	Phenol extraction and precipitation with ice cold ammonium acetate in methanol	2D Lysis buffer [urea 7 M, thiourea 2 M, CHAPS 4% (w/v)]	09
In-solution	Phenol extraction and precipitation with ice cold ammonium acetate in methanol	SDS buffer^a^ [0.5 M Tris-HCl pH 6.8, 50% (v/v) glycerol, 10% (w/v) SDS, 0.2 M DTT]	08
In-solution	Phenol extraction and precipitation with ice cold ammonium acetate in methanol	2D Lysis buffer + SDS buffer^a^ [urea 7 M, thiourea 2 M, CHAPS 4% (w/v), 0.5 M Tris-HCl pH 6.8, 50% (v/v) glycerol, 10% (w/v) SDS, 0.2 M DTT]	10
In-solution	TCA extraction and precipitation with 80% acetone (chilled)	2D Lysis buffer [urea 7 M, thiourea 2 M, CHAPS 4% (w/v)]	0
In-solution without precipitation	Boiling in SDS buffer^c^	50 mM ammonium bicarbonate	0
In-solution with Guanidium hydrochloride	Phenol extraction and precipitation with ice cold ammonium acetate in methanol	50 mM ammonium bicarbonate, 6 M Guanidium Hydrochloride	233
In-solution with Invitrosol	Boiling with SDS buffer^a^ and precipitated with 80% acetone (chilled)	50 mM ammonium bicarbonate, Invitrosol	888
In-solution without Invitrosol	Boiling with SDS buffer^a^ and precipitated with 80% acetone (chilled)	50 mM ammonium bicarbonate	814
In-solution with Rapigest	Boiling with SDS buffer^a^ and precipitated with 80% acetone (chilled)	50 mM ammonium bicarbonate, Rapigest	402
In-solution without Rapigest	Boiling with SDS buffer^a^ and precipitated with 80% acetone (chilled)	50 mM ammonium bicarbonate	720
In-solution with urea	Boiling with SDS buffer^b^ and precipitated with 80% acetone (chilled)	8 M urea	610

a,b,c *The SDS buffer composition is given in methods*.

For animal and bacterial tissues, FASP is widely used as it is a simple and efficient method (Wisniewski et al., 2009). In plants, only a few studies used FASP after extraction of protein using TCA/phenol (Wang et al., 2014; Zhang et al., 2015). We examined the applicability of FASP to tomato fruit tissue with an aim to simplify and reduce the number of steps involved in the sample preparation. After lysis of the homogenized fruit tissue in the SDS buffer^b^ on micron filters, followed by washing and digestion as per FASP protocol, no proteins were detected by LCMS analysis (data not shown). In contrast, repetition of the same procedure with protein obtained after phenol extraction and ammonium acetate precipitation followed by digestion on micron filters identified 1141 proteins (Figure 1). The above difference in protein identification emphasized the importance of phenolic extraction and precipitation prior to any subsequent treatments.

### Addition of surfactants has no appreciable impact on protein identification

Solubilization of proteins is essential to untangle the complex structural organization to simpler polypeptide chains by breaking the non-covalent interactions. Incomplete solubilization results in loss of proteins and a range of compounds such as chaotropes like urea, thiourea, detergents such as SDS, CHAPS (Molloy, 2000), and reducing agents like DTT or 2-mercaptoethanol (Mechin et al., 2003) are used in combination to assist protein solubilization. Recently, Chen et al. (2007) reported the usage of Invitrosol and Rapigest for improving the total proteome coverage in shotgun proteomics.

Table 1 shows an evaluation of both conventional chaotropes that are used in buffers as well as MS compatible surfactants for protein solubilization in in-solution digested samples. Usage of 8 M urea for solubilization of protein obtained after boiling with SDS buffer^b^ and precipitation with 80% acetone, yielded 610 proteins on separation on nanoLCMS. In contrast, use of 50 mM ammonium bicarbonate and/or guanidium hydrochloride/Invitrosol/Rapigest for dissolving proteins identified a total of 0, 233, 888, and 402 proteins respectively. The addition of Invitrosol though increased the number of proteins but not appreciably. The addition of Rapigest decreased the number of identified proteins (Table 1). Notwithstanding the inadequacy of Rapigest for tomato fruits, it has been used for microsomal protein extraction in tomato roots (Mbeunkui and Goshe, 2011). The solubilization of proteins obtained after phenol/TCA extraction in 2D lysis buffer/SDS buffer/and a combination of both was also inefficient, resulting in very few proteins (9, 8, 10, and 0 proteins) respectively (Table 1). Above results indicated that irrespective of the methods used for protein extraction and precipitation, the solubilization of proteins is a very critical step for obtaining good proteome coverage.

### Fractionation Is an essential step in proteome profiling of tomato fruits

**Figure 2:**
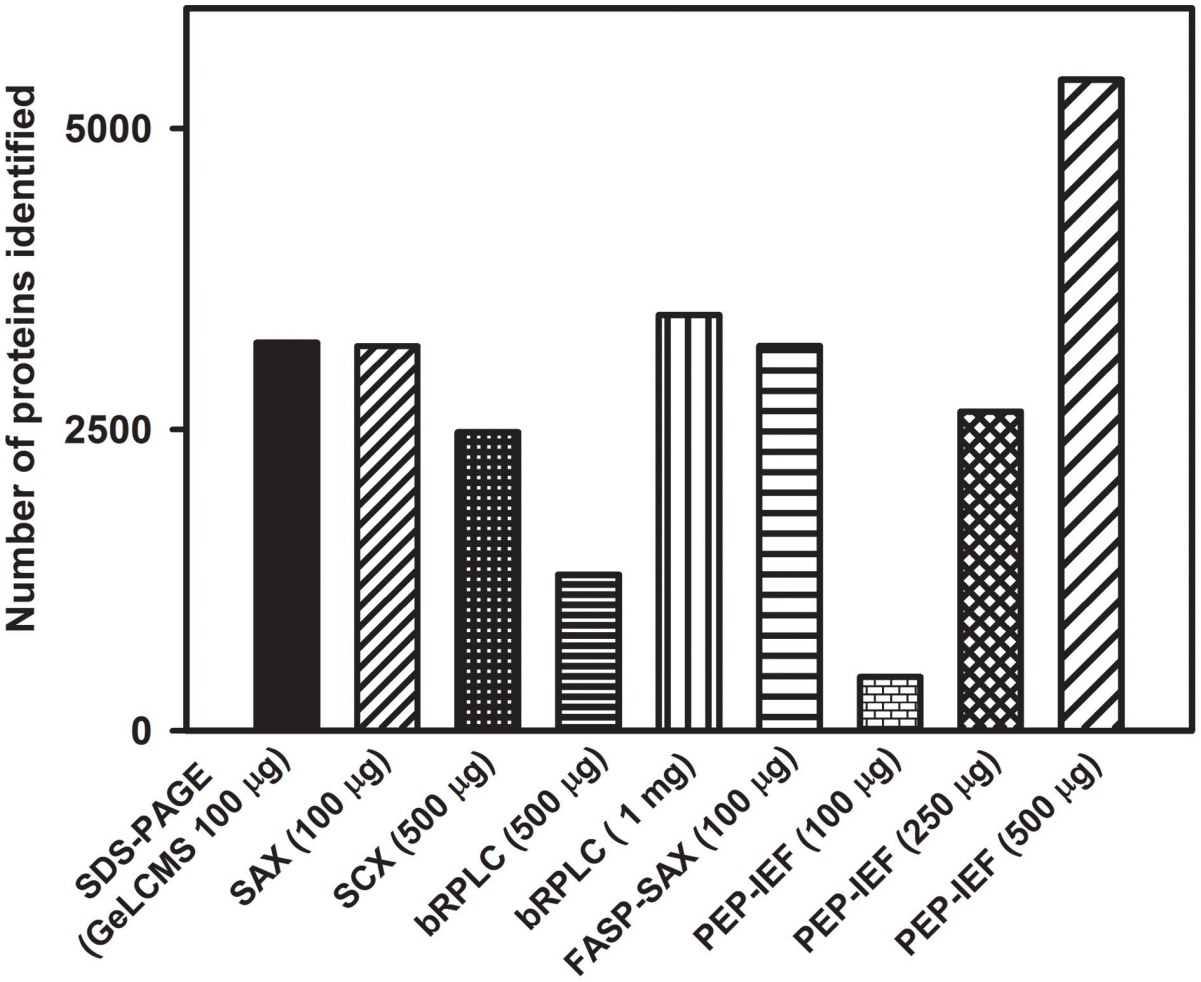
**Effect of different fractionation techniques on proteome coverage**. Only the proteins identified with ≥2 peptide matches are shown. The details of the buffer composition, fractionation conditions, etc. are described in methods.

Protein fractionation helps in reducing the inherent complexity of the sample and helps in improving the proteome coverage as well as the visibility of low abundance proteins. In the present study, we evaluated various fractionation techniques using the protein isolated from tomato fruits (Figure 2). Post-fractionation, peptides were separated on LC using different gradients based on the complexity of the sample and fractionation technique employed (Table S1). The size fractionation of proteins (100 μg) on SDS-PAGE followed by tryptic digestion resulted in the identification of 3220 (Phenol; Figures 1, 2) and 2907 (TCA; Figure 1) proteins after LCMS. Treatment of proteins obtained after phenol extraction with 6 M guanidium hydrochloride followed by tryptic digestion and further fractionation based on the charge by strong anion exchange chromatography (SAX) resulted in the identification of 3100 proteins (Figure 2). Replacement of SAX by SCX for protein separation considerably reduced the protein yield, identifying only 575 proteins starting from 100 μg protein (data not shown), whereas, when peptides obtained from 500 μg protein were loaded on SCX, 2477 proteins were obtained (Figure 2). This indicates that SAX has a better capability in yielding higher protein number with lower peptide load compared to SCX. It is plausible that the sulfopropyl Sepharose matrix used for SCX has a higher binding capacity, therefore no appreciable results were obtained on fractionation of peptides obtained from 100 μg protein. A similar opinion was also expressed by Mostovenko et al. (2013), where SCX gave better coverage on use of higher protein loads. However, when SAX was carried out using protein obtained from TCA/acetone, no proteins were identified. Similarly, FASP followed by separation of peptides on SAX resulted in 3195 proteins (Figure 2). FASP, when used alone, did not yield any protein identification implying the complexity of the protein mixture obtained from tomato fruit tissue and the importance of protein fractionation. A previous report revealed a high protein number starting with 75 μg peptide load on high pH RPLC in human peripheral blood mononuclear cells (Stein et al., 2013). However, when peptides obtained from 500 μg protein and 1 mg protein were fractionated on bRPLC, 1294, and 3452 proteins were obtained respectively suggesting that bRPLC works better at higher peptide loads at least in the case of tomato fruit extracts.

Fractionation of peptides obtained after digestion of 500 and 250 μg protein (obtained after phenol extraction) by peptide IEF (PEP-IEF) yielded about 5404 and 2649 proteins (Figure 2). On the contrary, PEP-IEF using an initial protein concentration of 100 μg (which was used for the previously mentioned in-gel and in-solution preparations) identified only 447 proteins. However, when PEP-IEF was performed using peptides obtained after digestion of 100 μg TCA-extracted protein, no proteins could be identified.

It is evident from the foregoing that GeLCMS and SAX (±FASP) using phenol-extracted sample resulted in higher proteome coverage, whereas, a 3–10-fold higher initial protein amount was required for a similar proteome coverage using PEP-IEF, SCX, or bRPLC. In essence, our results suggest that protein fractionation is essential for obtaining better proteome coverage, especially from tomato fruit tissue.

### Mass spectrometric parameters optimization

For shotgun proteome profiling, data-dependent acquisition (DDA) is the method of choice and involves a number of parameters that significantly affect the proteome coverage. Though a number of studies evaluated and optimized MS parameters focusing on *E. coli* and *S. cerevisiae* (Kim et al., 2010; Paulovich et al., 2010; Andrews et al., 2011; Kalli and Hess, 2012), there are no reports on optimization of MS parameters using plant tissues. In our study, we examined the most important MS parameters and their influence on proteome coverage in tomato fruits.

Based on the earlier reports (Kim et al., 2010; Paulovich et al., 2010; Andrews et al., 2011; Kalli and Hess, 2012), the number of settings examined for each MS parameter was narrowed down to two or three to avoid unnecessary iterations and redundancy (Table S2). All parameters were evaluated using the first fraction obtained from GeLCMS to maintain the uniformity. Taking cognizance of the parameters that gave encouraging protein identification, we finally validated the parameters by incorporating them into a DDA and checked their reproducibility using the fruits of different tomato cultivars.

### Automatic gain control (AGC) target and maximum ion injection time for MS and MS/MS events

AGC target regulates the ion population entering the mass analyzer (Kalli et al., 2013) while maximum ion injection/fill time defines the maximum time through which ions are allowed to fill in the ion trap (IT) or C-trap before they are transferred to Orbitrap (FT). These two parameters work inter-dependently, i.e., of these two parameters, whichever is first met by the instrument in a given time, triggers the successive MS or MS/MS event. Several AGC targets for MS and MS/MS events and ion injection times have been previously evaluated (Andrews et al., 2011; Kalli and Hess, 2012). In the current study, following AGC target values and fill times were evaluated in different combinations—in combination I, for MS, AGC target-fill time being 5E+05, 500 ms, and for MS/MS, 1E+04, 100 ms; in combination II, AGC target-fill time being 1E+06, 100 ms for MS, and 1E+04, 100 ms for MS/MS and in combination III, AGC target-fill time being 2E+06, 250 ms for MS and 3E+04, 200 ms for MS/MS event respectively.

**Figure 3:**
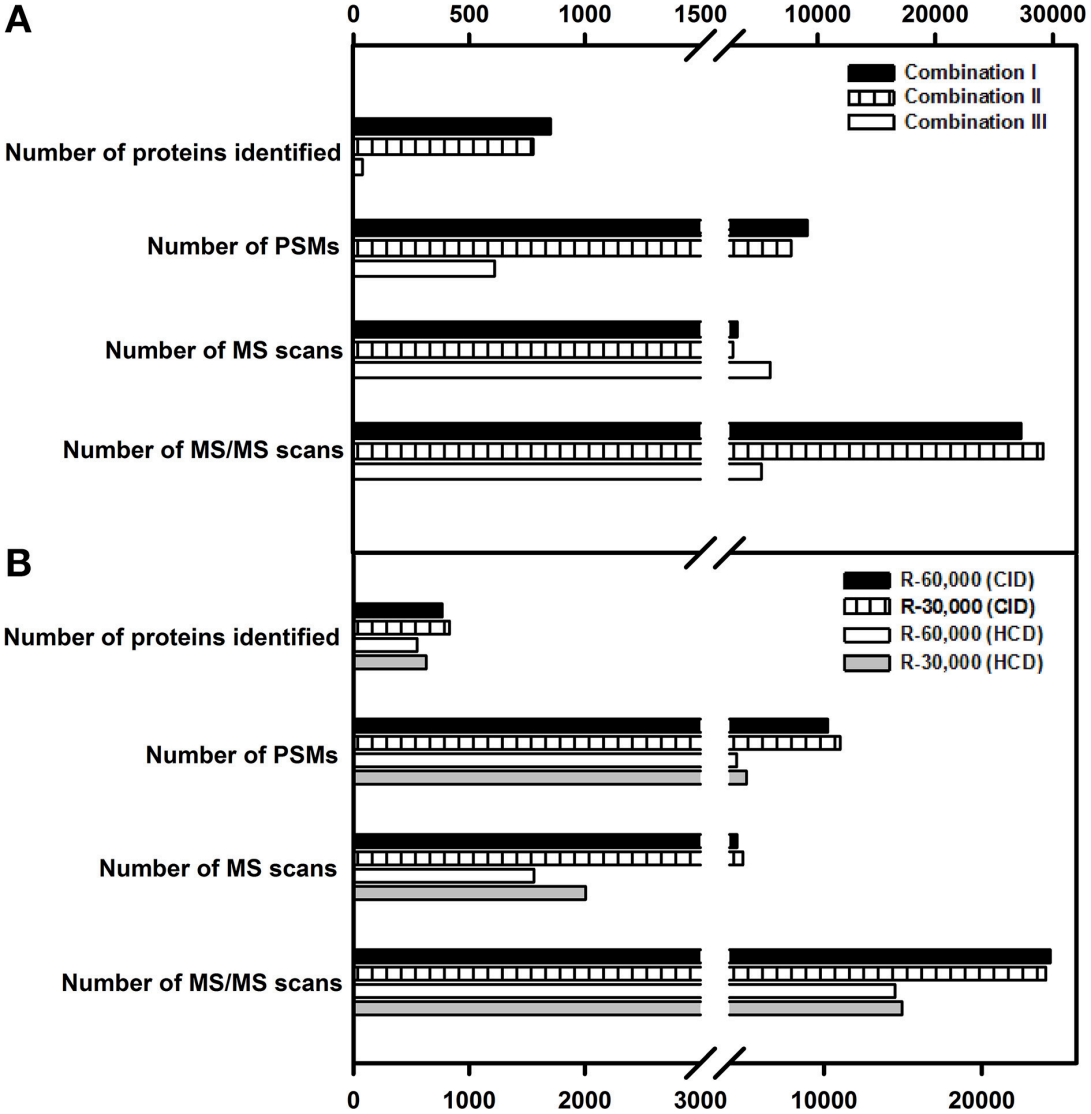
**Influence of AGC targets, fill times, resolution and fragmentation modes on protein identification. (A)** Combination I (AGC target values and fill times for MS-5E+05, 500 ms, and for MS/MS, 1E+04, 100 ms); Combination II (AGC target values and fill times for MS-1E+06, 100 ms and for MS/MS-1E+04, 100 ms); Combination III (AGC target values and fill times for MS-2E+06, 250 ms and for MS/MS-3E+04, 200 ms). **(B)** A resolution of 60,000 and 30,000 was examined for both CID and HCD fragmentation. Only the proteins identified with ≥2 peptide matches are shown.

Of these, combination I yielded the highest number of proteins of 852 and peptide spectral matches (PSM) of 9137 followed by combination II and combination III (Figure 3A). Combination III resulted in the least protein identification number and PSMs indicating the possibility of space charge effects due to the high target values.

### Resolving power and fragmentation mode

While increasing resolving power does improve the mass accuracy (Scigelova et al., 2011), it also increases the scan duration affecting the number of MS/MS spectra acquired. The effect of different combinations of FT-IT/FT-FT (FT-Orbitrap, IT-ion trap) on proteome profiling at different resolving powers were evaluated in great detail by Kim et al. (2010).

In the present study, given the bottom up proteome profiling, resolution at 60,000 and 30,000 for MS scans were evaluated for fragmentation (MS/MS) using both CID and HCD. While both the methods yield similar types of ions—b and y, the intensity of fragmentation differs (Page et al., 2005). In agreement with the earlier reported data (Kim et al., 2010), resolution of 30,000 yielded a greater number of PSMs and higher protein identification numbers in both CID and HCD modes (Figure 3B). The number of MS/MS events were higher at a survey scan resolution of 30,000 when compared to a resolution of 60,000. Also, the number of PSMs and proteins identified were greater in FT-IT combination than in FT-FT combination because of faster scan speeds and lower detection threshold in the ion trap, however, the overall spectral quality was better in HCD mode. While lesser resolution yielded increased protein identification, the difference in the number of proteins identified was not appreciably high.

### Monoisotopic precursor selection

**Figure 4:**
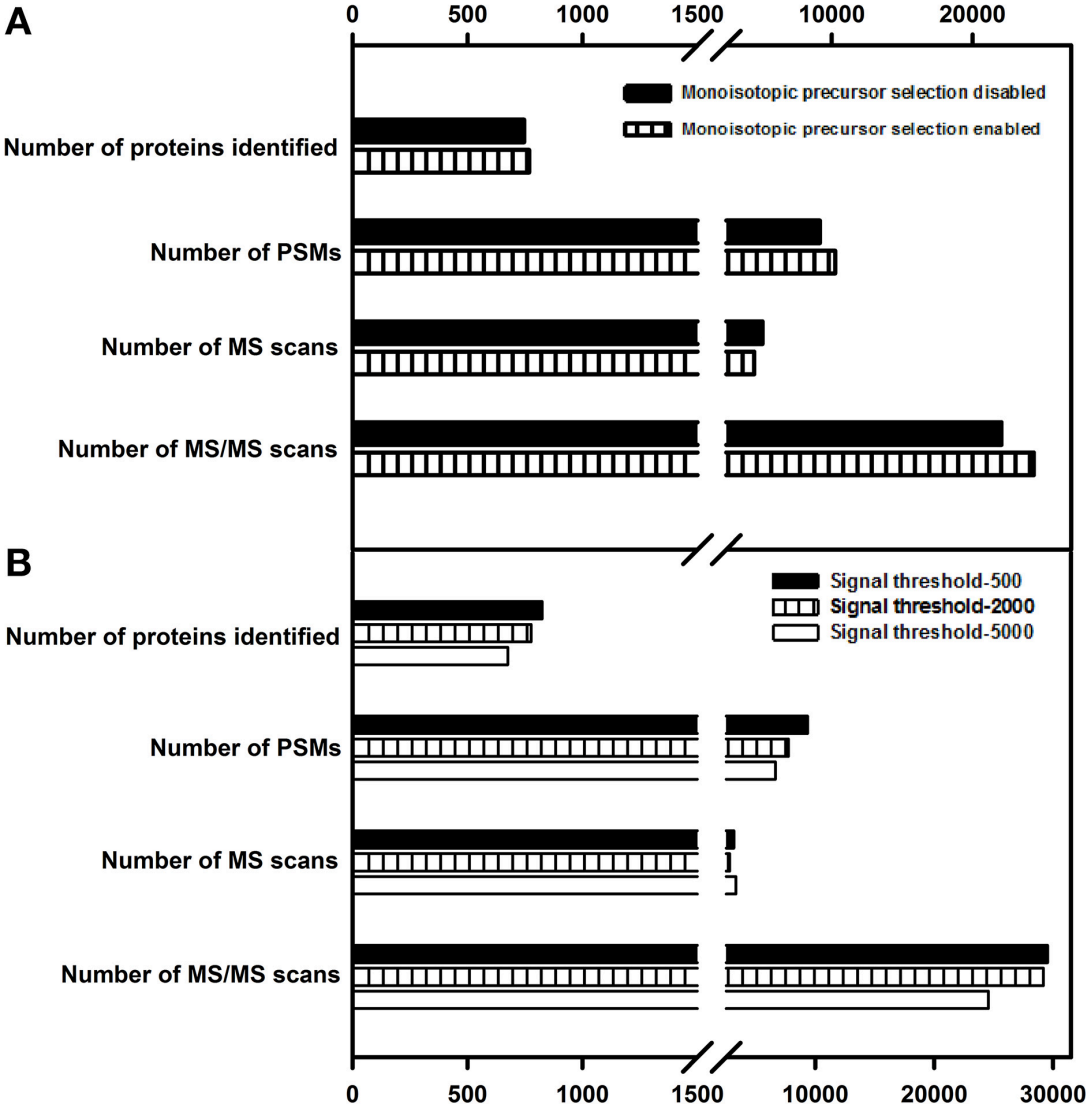
**Effect of monoisotopic precursor selection and signal threshold parameters on protein identification. (A)** Enabling or disabling the monoisotopic precursor ion selection was examined. **(B)** variation in signal threshold values at 500, 2000, and 5000 were examined. Only the proteins identified with ≥2 peptide matches are shown.

This parameter, when enabled in data dependent acquisition, causes fragmentation of the monoisotopic peak of the overall isotopic distribution, significantly improving the quality of peptide identification. Earlier studies (Andrews et al., 2011) suggested that enabling this parameter lead to a better proteome coverage. In the present study, enabling this parameter did not have any significant impact on the protein identification number; however, this parameter was enabled for further evaluations considering the quality of the data it yielded. Details of the number of MS and MS/MS events, PSMs and protein identification are listed in Figure 4A.

### Signal threshold

Signal threshold defines the minimum intensity required for a peak in MS scan to be chosen for fragmentation. Setting the signal threshold to a higher value improves the quality of the spectral data but reduces the number of MS/MS spectra (Wong et al., 2009). Also, when the dynamic range of proteins is large, the probability of identifying low abundance proteins decreases with increasing signal threshold value. In the current study, this parameter was evaluated for the following test values—500, 2000, and 5000. As stated above, the number of proteins identified decreased with increase in signal threshold. Details of the number of MS and MS/MS events, PSMs and protein identification are listed in Figure 4B. The value of signal threshold can be judiciously decided upon the basis of complexity of the sample, the type of proteome targeted and quality of spectra obtained.

### Top N

**Figure 5:**
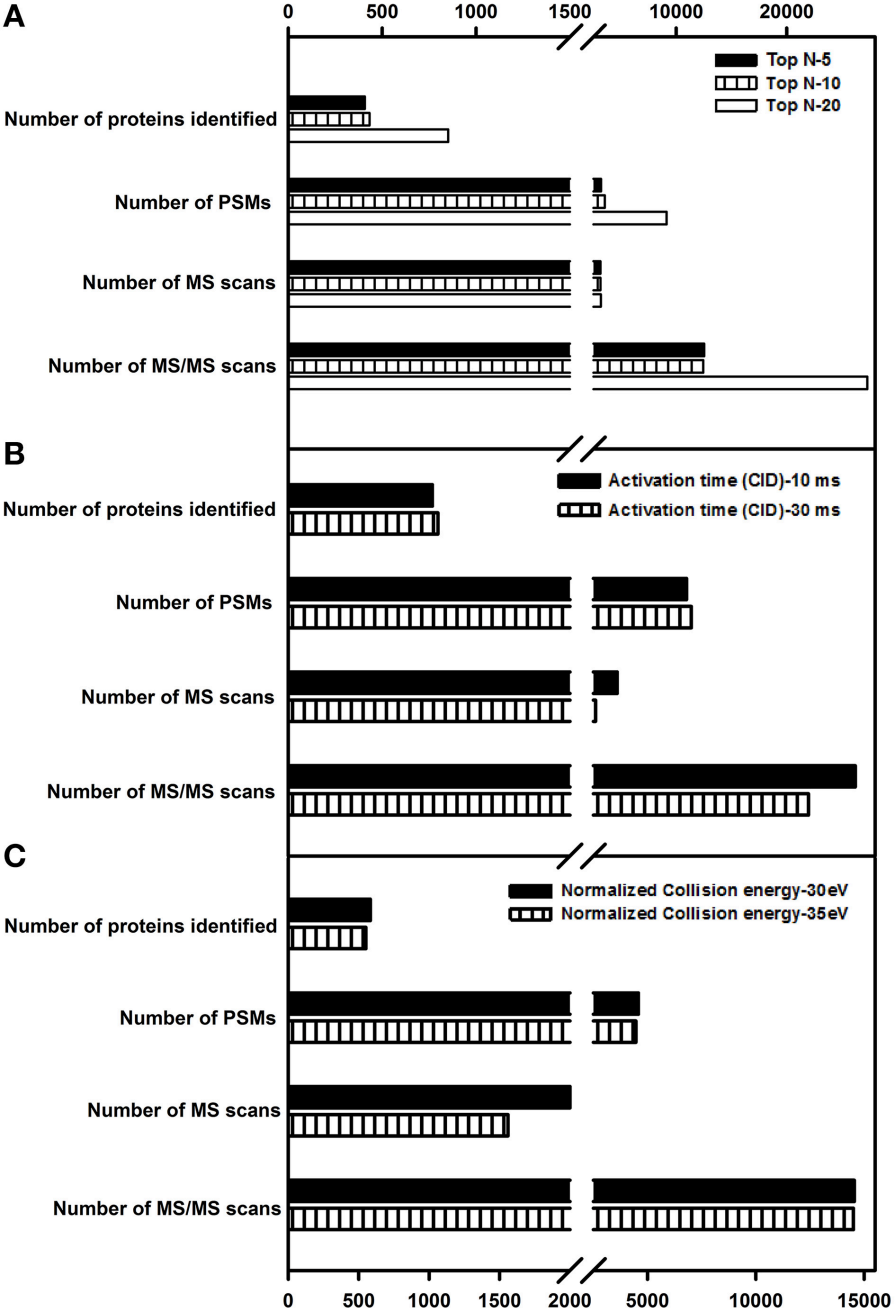
**Evaluation of Top *N*-value, activation time and activation energies on protein identification. (A)** A top *N*-value of 5, 10, and 20 was examined. **(B)** Two different activation times, 10 and 30 ms in CID fragmentation were checked. **(C)** Two different activation energies, 30 and 35 ev were examined for HCD fragmentation. Only the proteins identified with ≥2 peptide matches are shown.

This parameter defines the number of precursor ions that can be taken for fragmentation. Increasing this value increases the number of MS/MS spectra and the proteome coverage. However, an optimal value needs to be set as increasing the number of data-dependent scans increases the scan duration as well, in turn affecting the overall proteome coverage. In the current study, top *N*-values of 5, 10, and 20 were evaluated. Details of the number of MS and MS/MS events, PSMs and protein identification are listed in Figure 5A. In agreement with the earlier reports (Andrews et al., 2011; Kalli and Hess, 2012), the number of proteins identified decreased with the decrease in top *N*-value. A top *N*-value of 20 was found to be optimal for shotgun proteomics of tomato fruit tissue.

### Activation time and activation energy

Studies by Andrews et al. (2011) indicated that increase in activation time or energy did not have a drastic impact on protein identification number. In this study, activation time was evaluated with the following values- 10 and 30 ms for CID, while activation energies (Normalized Collision Energy-NCE) of 30 and 35 eV were evaluated for HCD to check for its impact on the protein identification. Similar to earlier studies (Andrews et al., 2011), there was a slight increase in the number of proteins identified, but no drastic difference was observed thereby alleviating the need for a longer activation time in CID. With respect to HCD, an increase in the activation energy had no significant increase in protein identification number. Details of the number of MS events, MS/MS events, PSMs, and protein groups identified for activation time (Figure 5B) and activation energy can be found in Figure 5C.

### Functional annotation of the proteins identified from different extraction and fractionation techniques

We further investigated whether the GeLC-MS approach was biased for proteins based on their molecular function, the biological processes they mediate or to the cellular component they belong. All the peptide fractionation techniques such as GeLCMS, SAX, FASP-SAX, PEP-IEF (250 μg), bRPLC (1 mg) and SCX that resulted in the identification of a similar number of proteins were considered for GO analysis. Overall, GO analysis revealed that none of the categories were significantly different between the peptide fractionation techniques employed in this study. However, membrane proteins were enriched marginally in SAX (33%) and FASP-SAX (36%) compared to GeLCMS (23%) (Figure S1). Moreover, for all the GO categories, the proportion of un-annotated proteins was lowest for GeLCMS than any other fractionation technique employed in this study.

Earlier reports indicate that FASP protocol extracts low abundance proteins (Wisniewski et al., 2009). In our study, we could identify proteins from low abundance classes comparable to FASP with GeLCMS, SCX, and bRPLC. From the foregoing, it is evident that GeLCMS is essentially unbiased as it did not lead to preferential extraction of proteins based on MW, charge, etc. (Schirle et al., 2003) and for any specific GO category.

### Validation of the peptide fractionation and MS parameters using different tomato cultivars

**Figure 6:**
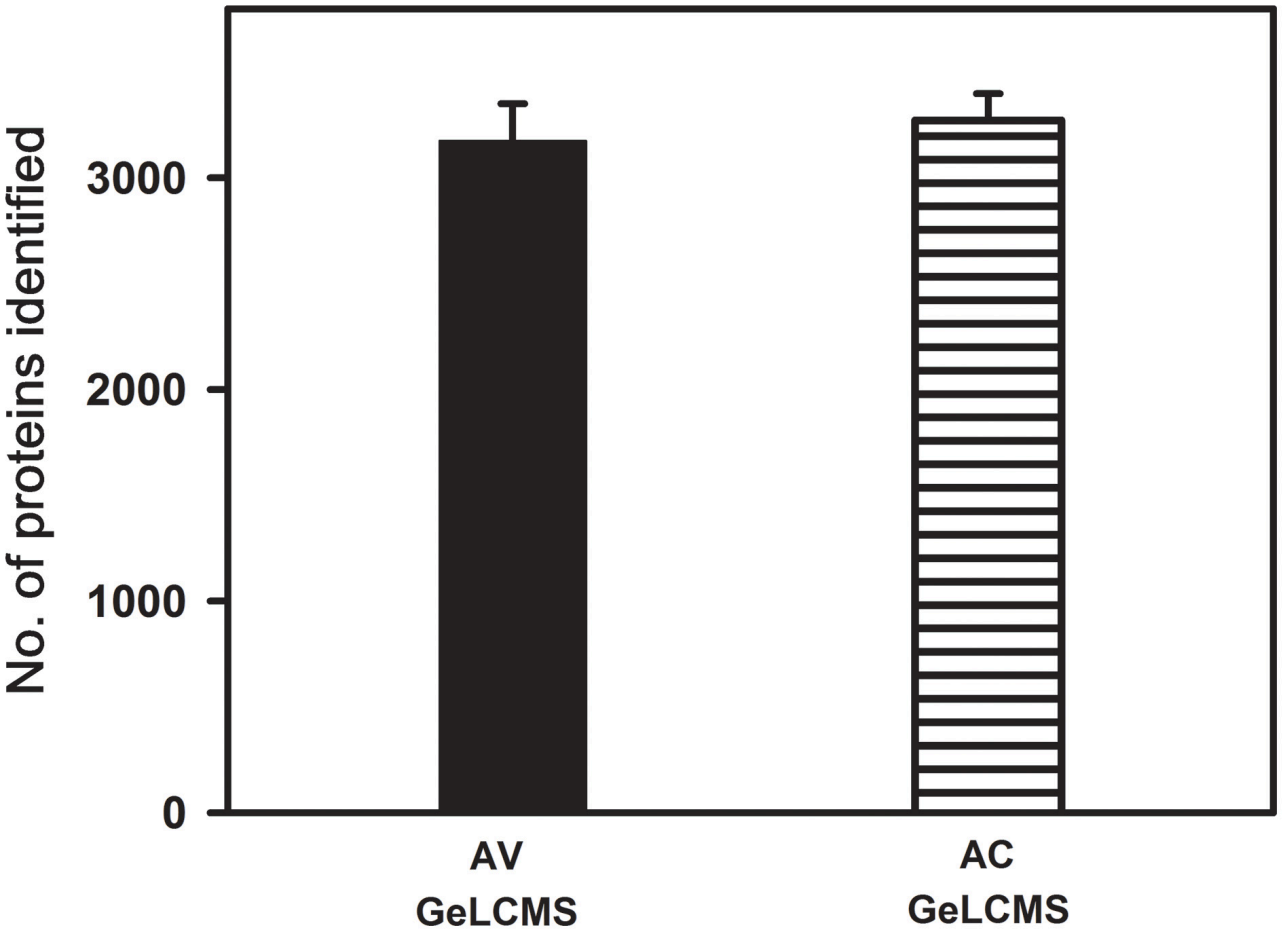
**Validation of optimized sample preparation and data dependent acquisition method using two tomato cultivars**. Proteins/peptides obtained after extraction with phenol and fractionation using GeLCMS from ripe fruits of tomato cultivars (AV and AC) were used for validation. Only the proteins identified with ≥2 peptide matches are shown (*n* = 3 ± SE).

To check the consistency of the evaluated MS parameters, the MS parameter values that gave encouraging results were incorporated into a DDA and applied to peptides obtained from in-gel samples from two different tomato cultivars and then subjected to LCMS analysis. The number of proteins identified were consistent in both the cultivars with CV < 10% suggesting the reproducibility and the robustness of the method (Figure 6). It is to be noted that the proteome coverage obtained in our study is much higher (3220 proteins) than reported earlier in tomato fruits using MUDPIT (Shah et al., 2012; 588 proteins) and 2D electrophoresis followed by protein identification by nLCMS (Xu et al., 2013; 506 proteins). As expected, subcellular fractionation and enrichment of chromoplasts from tomato fruits yielded a good proteome coverage with GeLCMS (1932 proteins- Barsan et al., 2012; 953 proteins-Wang et al., 2013). However, chromoplast isolation (Barsan et al., 2012; Wang et al., 2013) is time-consuming and requires large quantities of fruit tissue (a minimum of 250 g), whereas, our protocol uses only 1 g of fruit tissue as starting material.

Proteome coverage obtained in our study is also comparable to the data obtained in GeLCMS profiling of six human cell lines, wherein using 50 μg protein, 1785 proteins were identified (Schirle et al., 2003). Moreover, GeLCMS is very simple to use and cost effective compared to chromatography based approaches and can efficiently capture proteome encompassing a wide dynamic range. Also, the protocol can be scaled up depending on the experimental goals to obtain even better proteome coverage as PEP-IEF with 500 μg protein identified 5404 proteins.

### Cultivar-specific protein abundances in tomato fruits

#### GO analysis

We next examined the variation in the fruit proteome between two cultivars, AC, and AV using spectral counting of the proteins identified by GeLCMS. GO analysis revealed a similar proportion of functional categories of proteins based on their molecular function, biological process and cellular component in case of AC and AV suggesting no major differences in protein function in these two cultivars (Figure S2).

#### Identification of differentially expressed proteins between AC and AV fruits using label-free quantification

Of the total proteins identified in red ripe fruit samples of AV (3220) and AC (3272) (Figure 6), a total of 2664 and 2539 proteins could be quantified in a label-free manner using spectral counting in Scaffold (Table S3). The above quantification resulted in the identification of 315 differentially expressed proteins of which 235 proteins were upregulated, and 80 proteins were downregulated in AV in comparison to AC (Table S3). In addition, 123 proteins were detected only in AC whereas 215 proteins were detected only in AV (Table S4) with no single peptide hits of these proteins in the cultivars.

#### Functional classification of differentially expressed proteins

The differentially expressed proteins were classified into functional categories using Mapman (Usadel et al., 2009). All the upregulated proteins were classified into 36 functional classes, and 30 functional classes were identified in downregulated proteins. In both up- and downregulated proteins, protein degradation was the major category (8.8 and 11.3% respectively; Figure S3). Interestingly, 28 categories were observed in the proteins present only in AC and 34 categories among proteins present only in AV. In case of proteins that were identified only in AC, proteins belonging to “protein synthesis” formed the major class (12.2%; Figure S4). In AV, RNA regulation was the major category comprising of 7.6% of the proteins.

#### Protein complement mediating carotenoid metabolism and carotenoids are enriched in AV fruits

As tomato fruit is rich in carotenoids like lycopene and β-carotene, it was of interest to examine the carotenoid metabolism related proteins in the proteomes of AV and AC. Spectral counting revealed the presence of many of the carotenoid metabolism related proteins in AV proteome in our study. Phytoene desaturase (PDS; 2.1-fold) and ζ-carotene isomerase (ZISO; 10-fold), two of the enzymes involved in carotenoid biosynthesis were upregulated in AV and carotenoid cleavage dioxygenase 1B (CCD1B), a carotenoid degradation enzyme was 2.5-fold downregulated (Table S3). Interestingly, two of the carotenoid pathway enzymes including 1-deoxy-D-xylulose-5-phosphate reductoisomerase (DXR) and ζ-carotene desaturase (ZDS) were identified only in AV but not in AC (Table S4). This could be due to the low abundance of these proteins in AC fruits. Moreover, it has been previously reported that spectral counting is unable to detect the differences when the protein abundances are low (Liu et al., 2004; Old et al., 2005).

**Figure 7:**
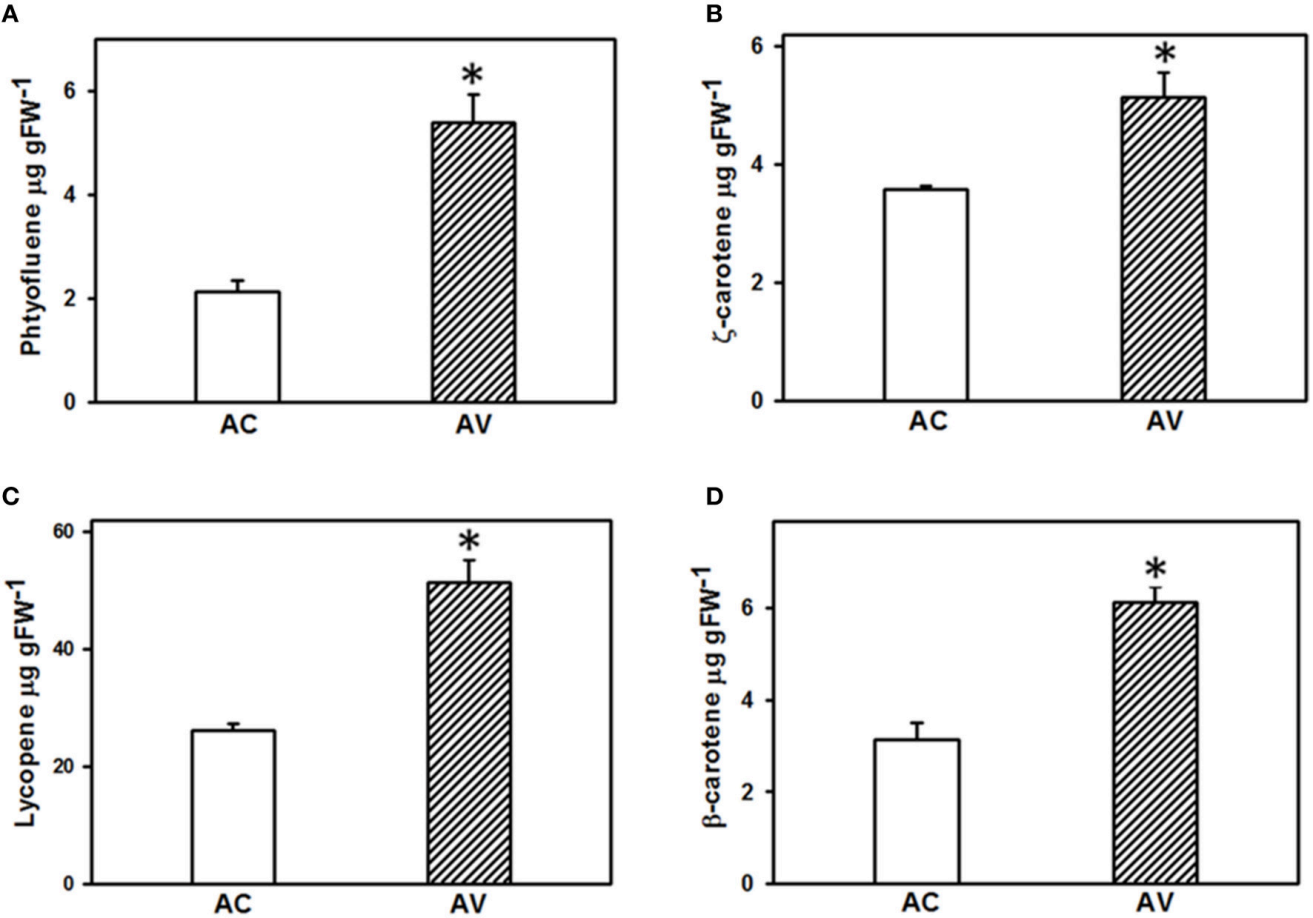
**Carotenoid profiling in the ripe fruits of AV and AC cultivars**. Carotenoid content was determined in the red ripe fruits of both AC and AV using the method described in (Gupta et al., 2015) (*n* = 3 ± SE). **(A)** Phytofluene; **(B)**, ζ-carotene; **(C)**, lycopene; **(D)**, β-carotene. ^*^Indicates significant differences with *P* ≤ 0.05.

To elucidate the correlation between the abundance of proteins related to carotenoid metabolism and carotenoid content, carotenoid profiles in the fruits of both AC and AV cultivars were determined and compared. Out of eight carotenoids identified in AC and AV, only four carotenoids, phytofluene, ζ-carotene, lycopene, and β-carotene were significantly different between both the cultivars (Figure 7). Among all the carotenoids, lycopene is the major carotenoid which was 1.96-fold higher in AV compared to AC fruits. Phytofluene (2.5-fold), ζ-carotene (1.5-fold), and β-carotene (1.95-fold) also accumulated in AV compared to AC, contributing to the high carotenoid content in AV fruits. The upregulation of enzymes mediating carotenoid biosynthesis and downregulation of carotenoid degradation enzymes in AV proteome highly correlates with the elevated carotenoid levels in AV fruits and also explains the dark red fruit phenotype of AV in comparison to AC fruits.

## Conclusions

An optimal protocol for proteome isolation and identification from tomato fruits was developed by systematic evaluation of various sample preparation protocols and MS parameters. For sample preparation from tomato fruits, protein precipitation was an essential and indispensable step. The usage of buffer saturated phenol most efficiently extracted proteins followed by TCA. The protein fractionation was best achieved using GeLCMS. For in-solution samples, SAX was a better fractionation technique yielding good proteome coverage. Considering that a minimal amount of protein (100 μg) gave a five-fold high proteome coverage, GeLCMS/SAX has potential to be used for the samples with limited amount of tissue.

To our knowledge, this is the first study evaluating various MS parameters for proteome profiling of tomato fruits. Among the parameters evaluated, AGC target, ion injection times, resolution and signal threshold had a major influence on proteome coverage. Though conventionally CID is the most widely used fragmentation mode for proteome profiling, usage of HCD resulted in better MS/MS data. The choice between CID vis-a-vis HCD can be made as per user discretion based on the desired experimental goals. The optimization of parameters reported in this study can serve as a starting point for similar optimization for other fleshy fruits. It is apt to note that MS parameter settings described here would vary for other systems owing to the heterogeneity in instrumentation platforms and nature of the biological matrix from which proteome profiling is desired. The outlined protocols can be used for high-throughput analysis of tomato fruit samples and can be optimized for shotgun proteome profiling of other plant tissues.

## Compliance with ethical standards

This article does not contain any studies with human participants or animals performed by any of the authors.

## Author contributions

The study was conceptualized by HK, KM, RS, and YS. VM standardized GeLCMS, surfactant treatments, HK performed all protein extractions, solubilizations, fractionation, validation, and carotenoid profiling, KM performed MS optimization, gradient optimization on LC-MS and in-solution digestions using TCA, HS performed SCX and bRPLC, HK, KM, HS, and YS analyzed the data, HK and HS maintained the plants and collected tissue. KM, HK, HS, RS, and YS wrote the manuscript. All authors read and approved the manuscript.

### Conflict of interest statement

The authors declare that the research was conducted in the absence of any commercial or financial relationships that could be construed as a potential conflict of interest.

## Abbreviations

DDA: Data Dependent Acquisition
PSM: Peptide Spectral Match
AGC: Automatic Gain Control
NCE: Normalized Collision Energy
HCD: Higher energy C-trap Dissociation
GeLCMS: 1D SDS-PAGE followed by LCMS.
