# Supplementary material for: Shotgun Proteomics of Tomato Fruits: Evaluation, Optimization and Validation of Sample Preparation Methods and Mass Spectrometric Parameters
Source: Front Plant Sci. 2016 Jun 29;7:969. doi: 10.3389/fpls.2016.00969 (PMC4925719; doi:10.3389/fpls.2016.00969)
Supplement: Supplementary file 1 [file Table1.PDF]

Table S1. Details of gradient used for different fractionation techniques. Shorter run times were used wherever multiple fractions were obtained as in the case of SCX, bRPLC or PEP-IEF.

Fractionation technique	Run time	Gradient employed
In-gel (GeLCMS)	118 min	5%-30% B (0-100 min), 30%-95% B (101-103 min), 95% B hold (104-106 min), 95%-5% B (107-113 min), 5% B hold (114-118 min)
SAX	100 min	8%-45% B (0-82 min), 45%-95% B (83-85 min), 95% B hold (86-88 min), 95%-5% B (89-95 min), 5% B hold (96-100 min)
SCX	52 min	0% -100% B (0-50 min), 5% B (52 min)
bRPLC	42 min	90%-10% B (0-39 min), 60%-40% B (40-41 min), 90%-10% B (42 min)
FASP-SAX	95 min	5%-8% B (0-10 min), 8%-18% B (11-25 min), 18%-25% B (26-40 min), 25-32% B (41-55 min), 32%-40% B (56-65 min), 40%-52% B (66-70 min), 52%-95% B (71-78 min), 95% B hold (79-85 min), 95%-5% B (86-88 min), 5% B hold (89-95 min)
PEP-IEF, FASP-PEP-IEF	60 min	5%-30% B (0 -48 min), 30%-95% B (49-53 min), 95% B hold (54-56 min), 95%-5% B (57-58 min), 5% B hold (59-60 min)
In-solution	150 min	5%-30% B (0-128 min),30%-95% B (129-131 min), 95% B hold (132-138 min), 95%-5% B (139-145 min), 5% B hold (146-150 min)
