# Supplementary material for: Shotgun Proteomics of Tomato Fruits: Evaluation, Optimization and Validation of Sample Preparation Methods and Mass Spectrometric Parameters
Source: Front Plant Sci. 2016 Jun 29;7:969. doi: 10.3389/fpls.2016.00969 (PMC4925719; doi:10.3389/fpls.2016.00969)
Supplement: Supplementary file 2 [file Table2.PDF]

Table S2. List of data dependent parameters evaluated in MS optimization

MS parameter	Settings evaluated
AGC target for MS event (FT)	5E+05, 1E+06, 2E+06
AGC target for MS/MS event (IT)	1E+04, 3E+04
Maximum injection time for MS (milliseconds)	100, 250, 500
Maximum injection time for MS/MS (milliseconds)	100, 200
Resolution	30000, 60000
Monoisotopic precursor selection	enabled, disabled
Minimum signal threshold	500, 1000, 2000
Top N	5, 10, 20
Activation time (milliseconds)	10, 30
Activation energy (Normalized Collision Energy)	30,35
Fragmentation mode	CID, HCD
