# Supplementary material for: Shotgun Proteomics of Tomato Fruits: Evaluation, Optimization and Validation of Sample Preparation Methods and Mass Spectrometric Parameters
Source: Front Plant Sci. 2016 Jun 29;7:969. doi: 10.3389/fpls.2016.00969 (PMC4925719; doi:10.3389/fpls.2016.00969)
Supplement: Supplementary file 3 [file Presentation1.PDF]

Shotgun Proteomics of Tomato Fruits: Evaluation, Optimization and Validation of Sample Preparation Methods and Mass Spectrometric Parameters

Himabindu Vasuki Kilambi[#], Kalyani Manda[#], Hemalatha Sanivarapu, Vineet Kumar Maurya, Rameshwar Sharma and Yellamaraju Sreelakshmi*

Repository of Tomato Genomics Resources, Department of Plant Sciences, School of Life Sciences, University of Hyderabad, Hyderabad-500046, India

[#]equal contribution by these authors

***Corresponding author at:** Repository of Tomato Genomics Resources, Department of Plant Sciences, University of Hyderabad, Hyderabad-500046, India. Tel: +91-40-23134771, Fax: +91-40-23010120.

Authors' email address

HVK, h.vasuki2@gmail.com, KM, nidhimanda19@gmail.com, HS, hemalatha.adr@gmail.com, VKM, vineetkm2000@gmail.com, RS rameshwar.sharma@gmail.com, YS, syellamaraju@gmail.com.

Running head: Shotgun Proteomics of Tomato Fruits

A

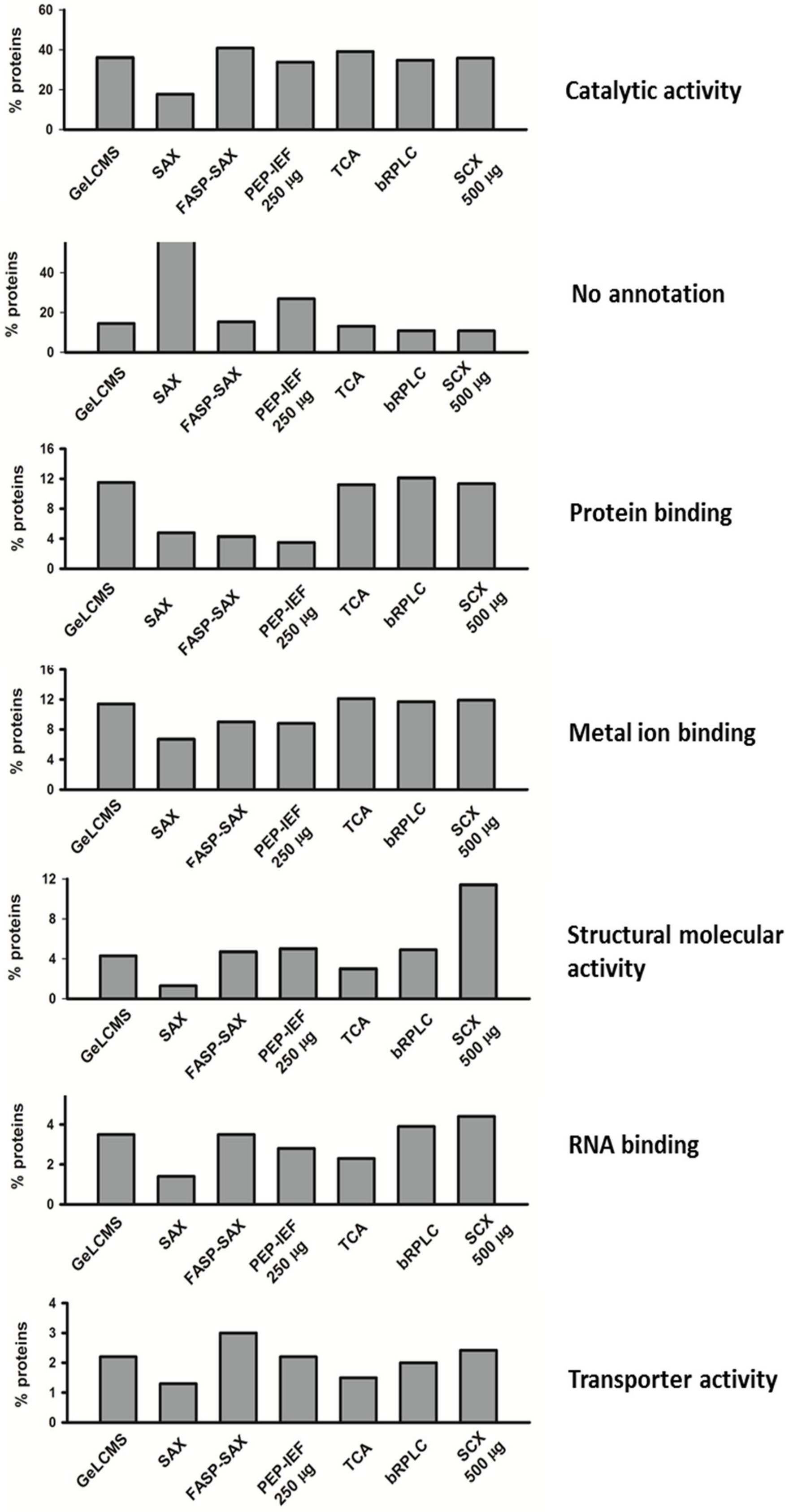

% proteins
GeLCMS
SAX
FASP-SAX
PEP-IEF 250 µg
TCA
bRPLC
SCX 500 µg
Catalytic activity
No annotation
Protein binding
Metal ion binding
Structural molecular activity
RNA binding
Transporter activity

Figure S1

A

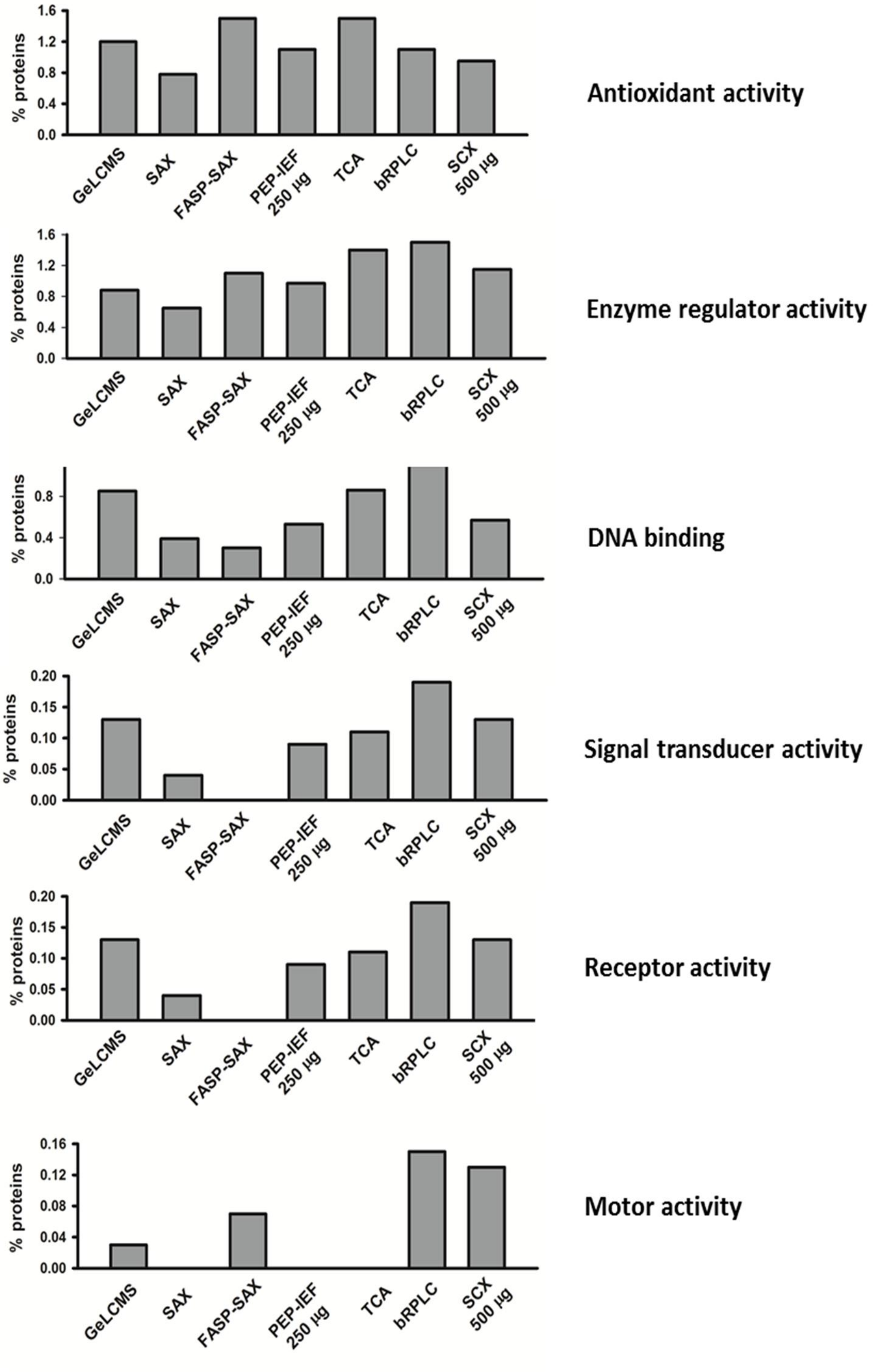

% proteins
GeLCMS
SAX
FASP-SAX
PEP-IEF 250 µg
TCA
bRPLC
SCX 500 µg
Antioxidant activity
Enzyme regulator activity
DNA binding
Signal transducer activity
Receptor activity
Motor activity

Figure S1

B

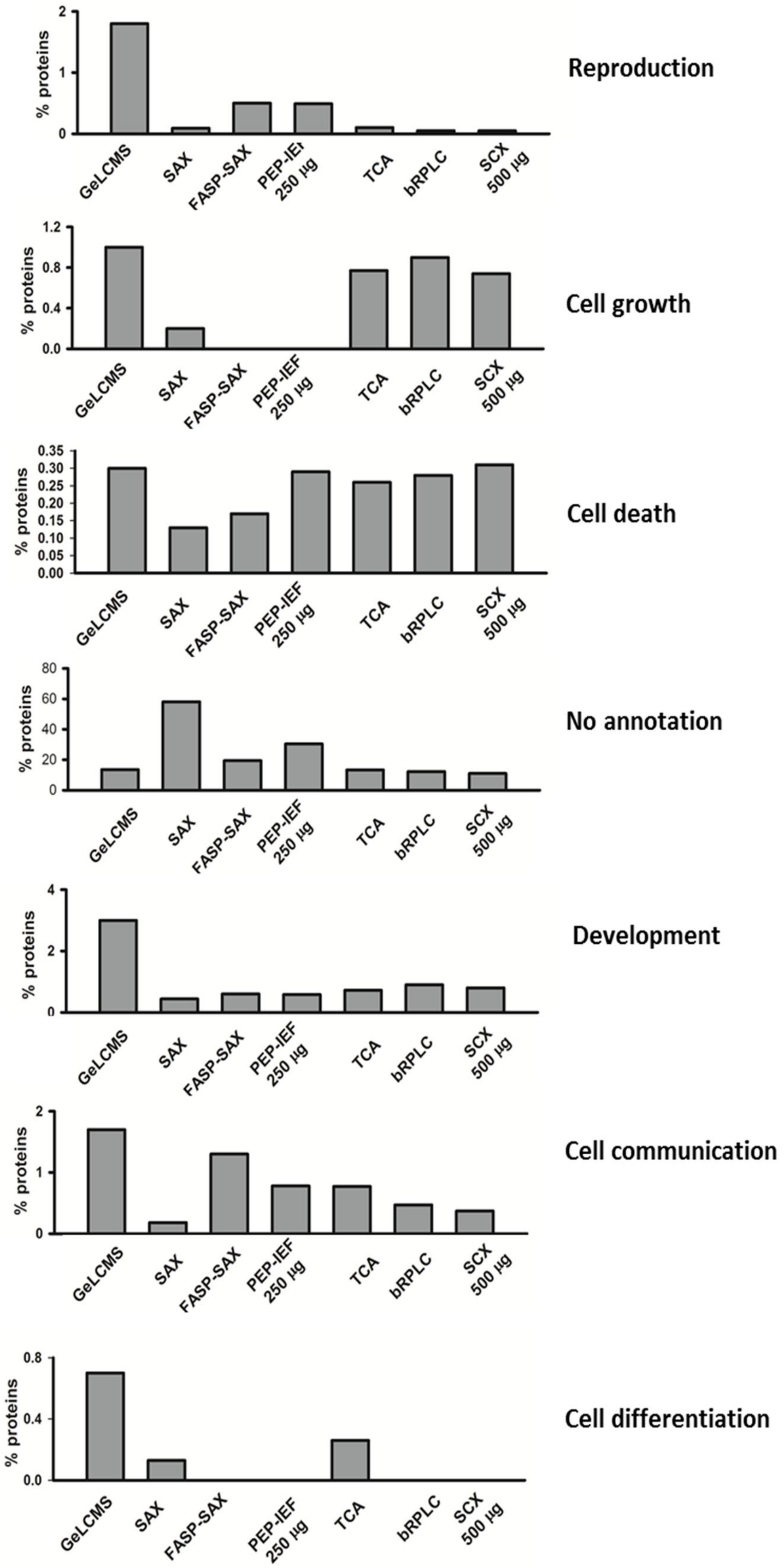

Reproduction
Cell growth
Cell death
No annotation
Development
Cell communication
Cell differentiation
% proteins
GeLCMS
SAX
FASP-SAX
PEP-IEF
250 µg
TCA
bRPLC
SCX
500 µg

Figure S1

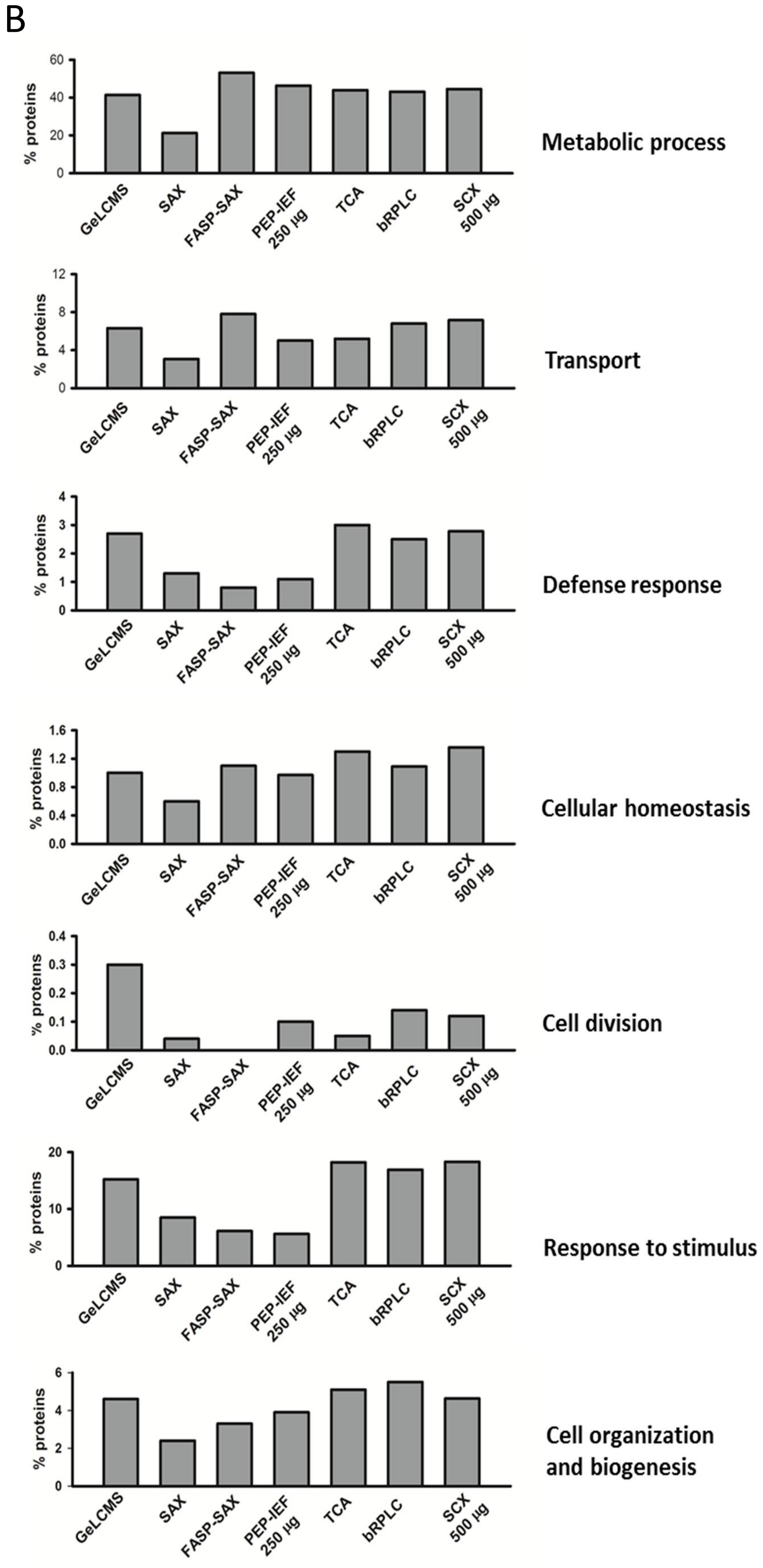
B
% proteins
GeLCMS
SAX
FASP-SAX
PEP-IEF 250 µg
TCA
bRPLC
SCX 500 µg
Metabolic process
Transport
Defense response
Cellular homeostasis
Cell division
Response to stimulus
Cell organization and biogenesis

Figure S1

B

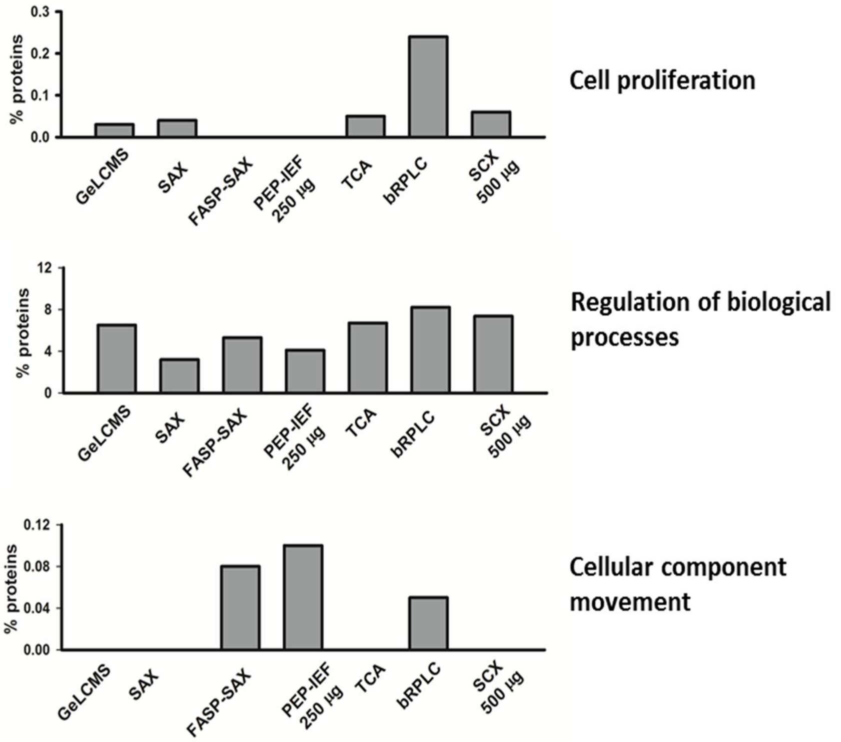

% proteins
0.3
0.2
0.1
0.0
GeLCMS
SAX
FASP-SAX
PEP-IEF 250 µg
TCA
bRPLC
SCX 500 µg
Cell proliferation
12
8
4
0
Regulation of biological processes
0.12
0.08
0.04
0.00
Cellular component movement

Figure S1

C

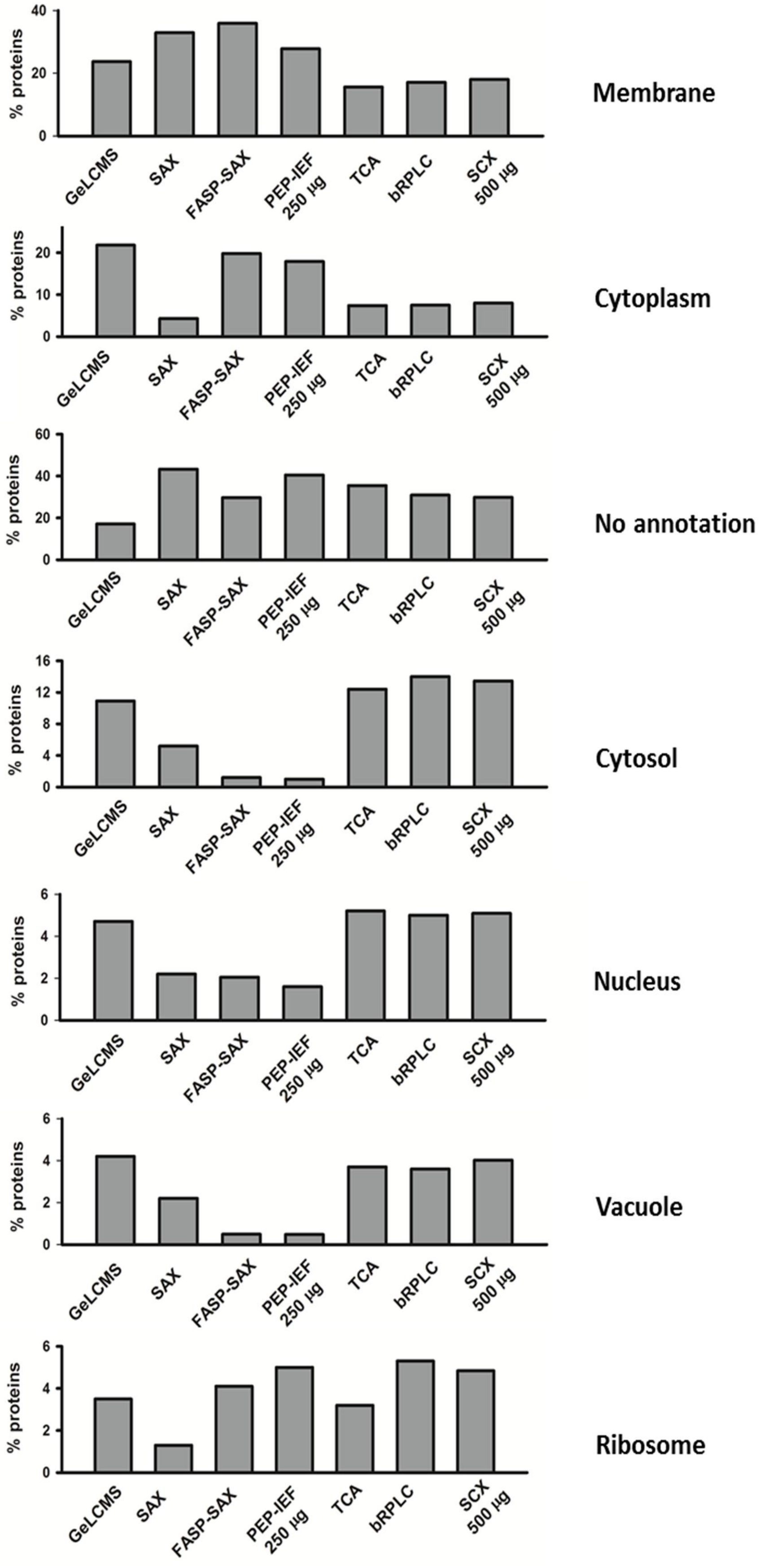
% proteins
0
20
40
GeLCMS
SAX
FASP-SAX
PEP-IEF 250 µg
TCA
bRPLC
SCX 500 µg
Membrane
% proteins
0
10
20
Cytoplasm
% proteins
0
20
40
60
No annotation
% proteins
0
4
8
12
16
Cytosol
% proteins
0
2
4
6
Nucleus
% proteins
0
2
4
6
Vacuole
% proteins
0
2
4
6
Ribosome

Figure S1

C

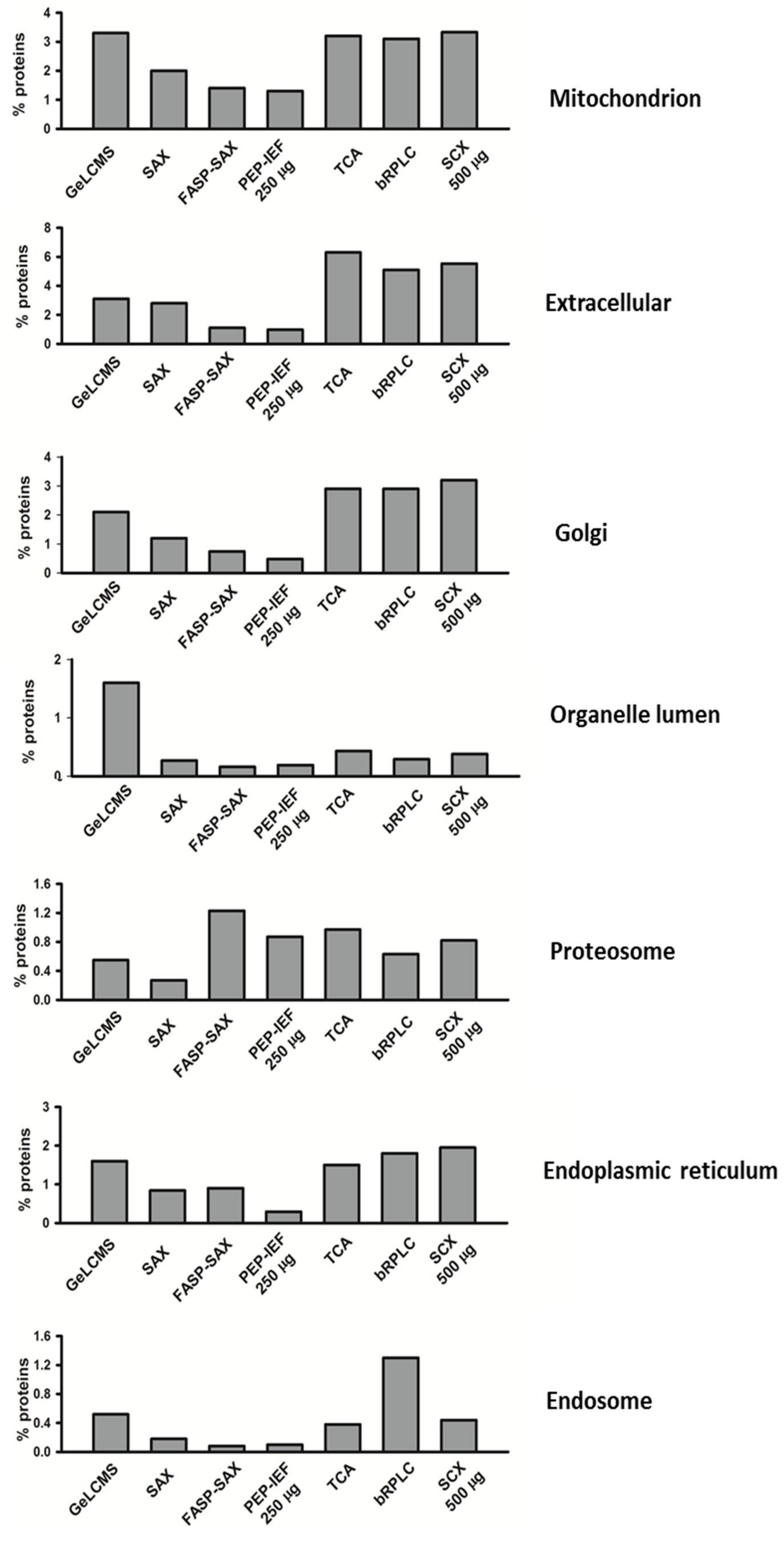
% proteins
GeLCMS
SAX
FASP-SAX
PEP-IEF 250 µg
TCA
bRPLC
SCX 500 µg
Mitochondrion
Extracellular
Golgi
Organelle lumen
Proteosome
Endoplasmic reticulum
Endosome

Figure S1

C

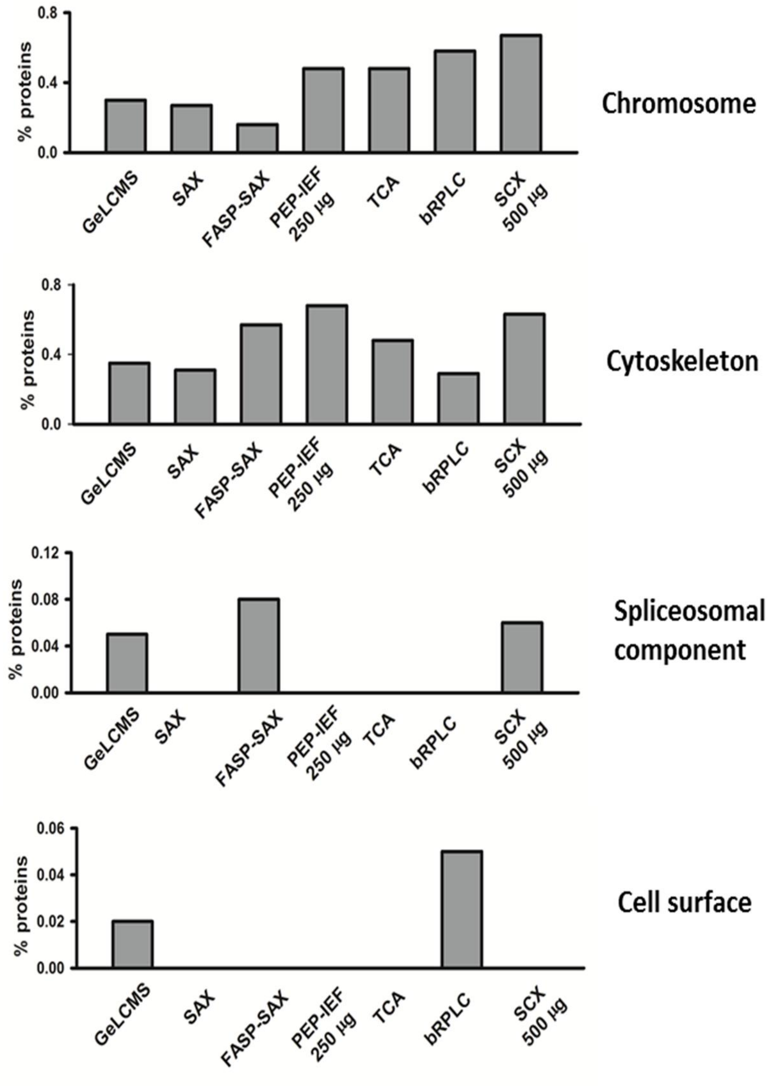

Figure S1. GO annotation of the proteins identified using different fractionation methods **A**, based on molecular function **B**, based on biological process, **C**, based on cellular component.
