# Supplementary material for: Shotgun Proteomics of Tomato Fruits: Evaluation, Optimization and Validation of Sample Preparation Methods and Mass Spectrometric Parameters
Source: Front Plant Sci. 2016 Jun 29;7:969. doi: 10.3389/fpls.2016.00969 (PMC4925719; doi:10.3389/fpls.2016.00969)
Supplement: Supplementary file 4 [file Presentation2.PDF]

Shotgun Proteomics of Tomato Fruits: Evaluation, Optimization and Validation of Sample Preparation Methods and Mass Spectrometric Parameters

Himabindu Vasuki Kilambi[#], Kalyani Manda[#], Hemalatha Sanivarapu, Vineet Kumar Maurya, Rameshwar Sharma and Yellamaraju Sreelakshmi*

Repository of Tomato Genomics Resources, Department of Plant Sciences, School of Life Sciences, University of Hyderabad, Hyderabad-500046, India

[#]equal contribution by these authors

***Corresponding author at:** Repository of Tomato Genomics Resources, Department of Plant Sciences, University of Hyderabad, Hyderabad-500046, India. Tel: +91-40-23134771, Fax: +91-40-23010120.

Authors' email address

HVK, h.vasuki2@gmail.com, KM, nidhimanda19@gmail.com, HS, hemalatha.adr@gmail.com, VKM, vineetkm2000@gmail.com, RS rameshwar.sharma@gmail.com, YS, syellamaraju@gmail.com.

Running head: Shotgun Proteomics of Tomato Fruits

A

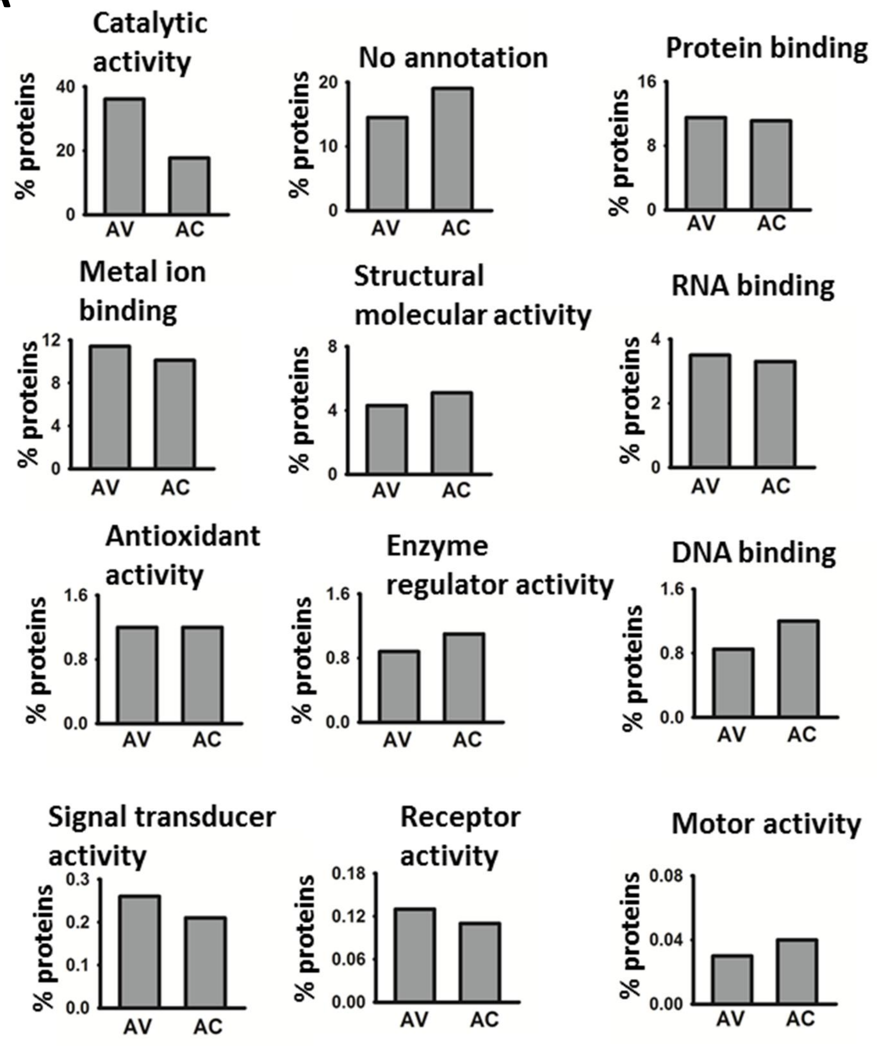
Catalytic activity
% proteins
40
20
0
AV
AC
No annotation
% proteins
20
10
0
AV
AC
Protein binding
% proteins
16
8
0
AV
AC
Metal ion binding
% proteins
12
8
4
0
AV
AC
Structural molecular activity
% proteins
8
4
0
AV
AC
RNA binding
% proteins
4
2
0
AV
AC
Antioxidant activity
% proteins
1.6
0.8
0.0
AV
AC
Enzyme regulator activity
% proteins
1.6
0.8
0.0
AV
AC
DNA binding
% proteins
1.6
0.8
0.0
AV
AC
Signal transducer activity
% proteins
0.3
0.2
0.1
0.0
AV
AC
Receptor activity
% proteins
0.18
0.12
0.06
0.00
AV
AC
Motor activity
% proteins
0.08
0.04
0.00
AV
AC

Figure S2

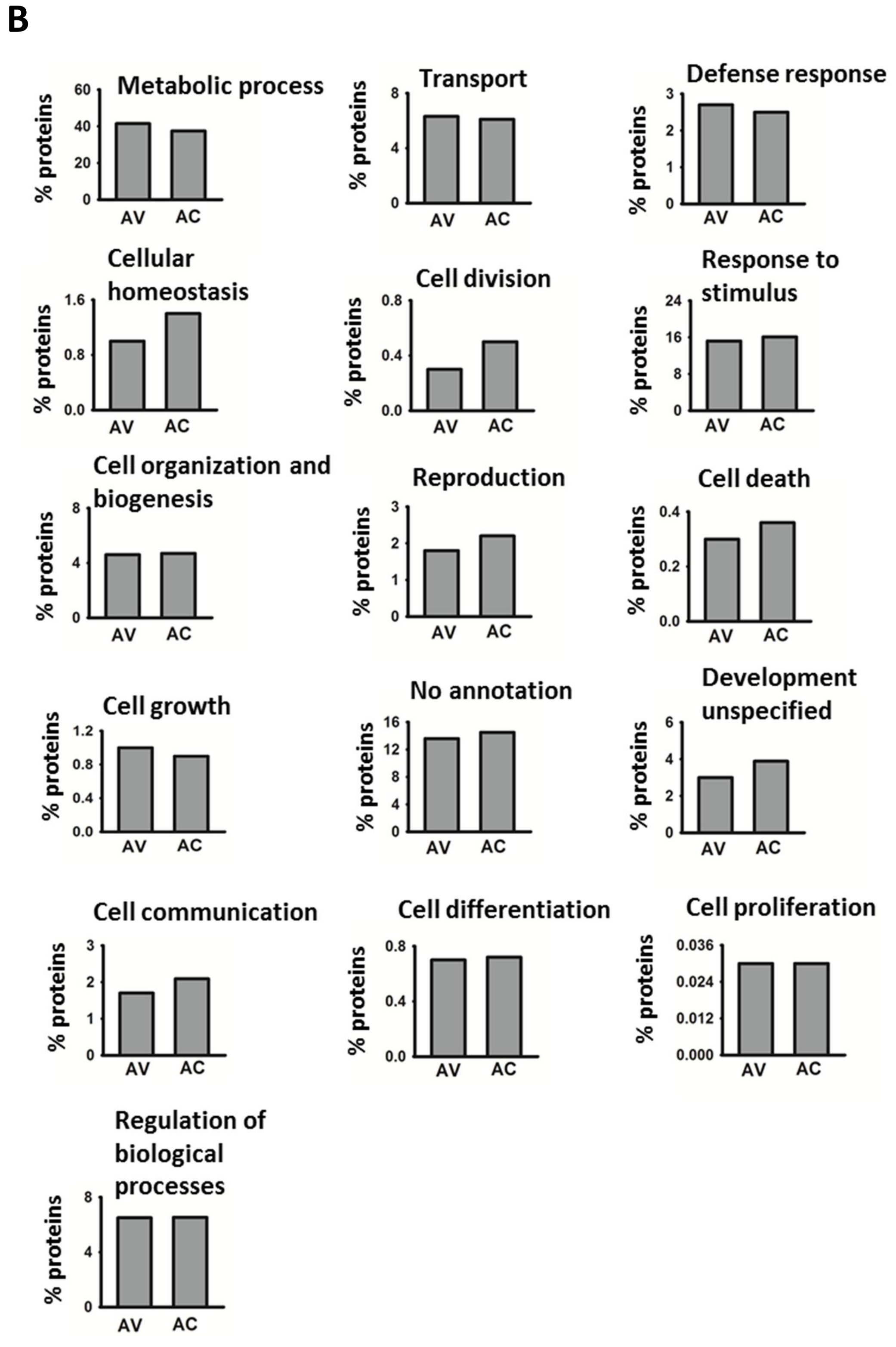
B
Metabolic process
Transport
Defense response
Cellular homeostasis
Cell division
Response to stimulus
Cell organization and biogenesis
Reproduction
Cell death
Cell growth
No annotation
Development unspecified
Cell communication
Cell differentiation
Cell proliferation
Regulation of biological processes
% proteins
AV
AC

Figure S2

C

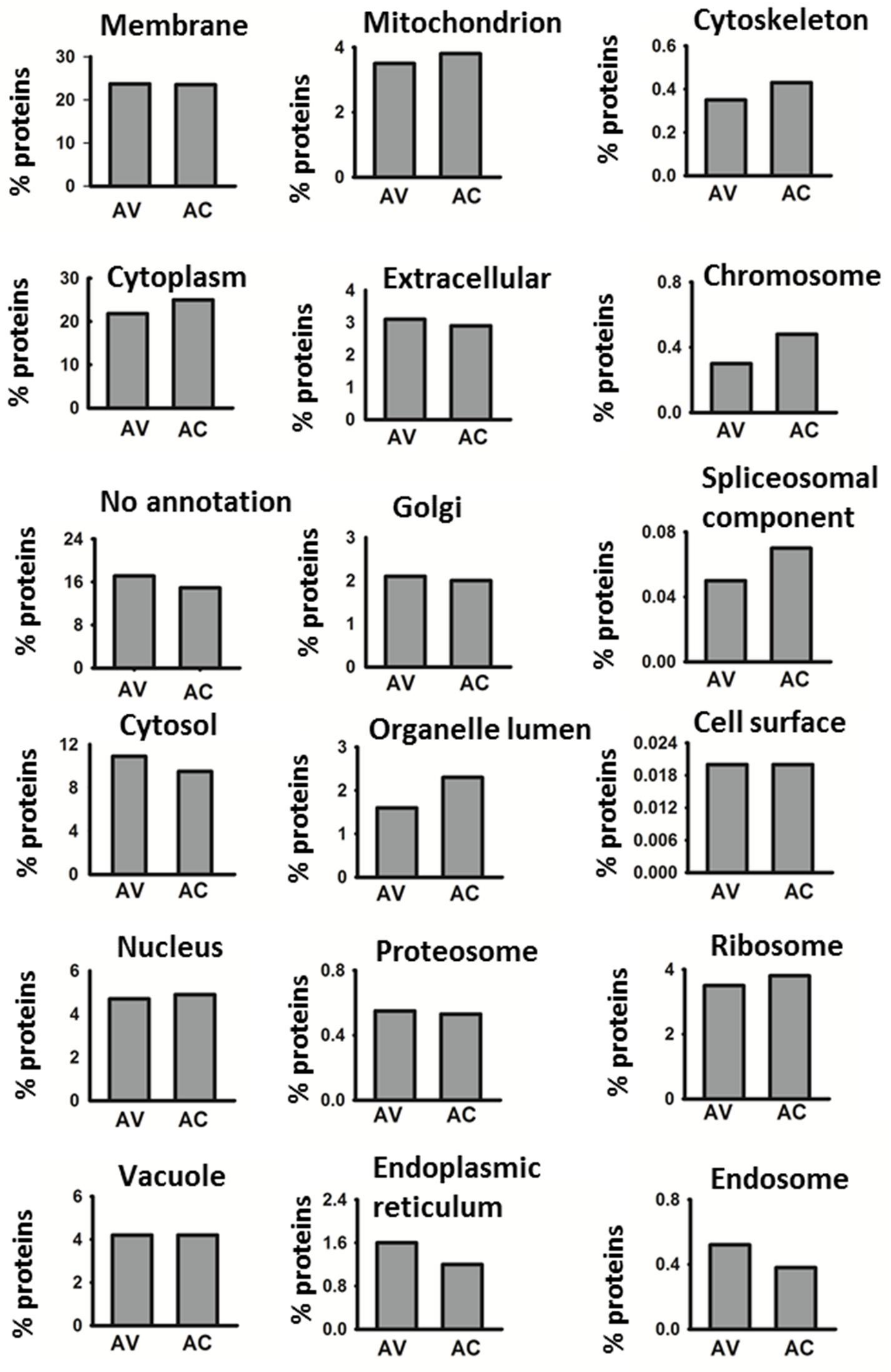

Figure S2. GO annotation of the proteins identified in AC and AV cultivars A, based on molecular function, B, based on biological process, C, based on cellular component.
