# Supplementary material for: Shotgun Proteomics of Tomato Fruits: Evaluation, Optimization and Validation of Sample Preparation Methods and Mass Spectrometric Parameters
Source: Front Plant Sci. 2016 Jun 29;7:969. doi: 10.3389/fpls.2016.00969 (PMC4925719; doi:10.3389/fpls.2016.00969)
Supplement: Supplementary file 5 [file Presentation3.PDF]

Shotgun Proteomics of Tomato Fruits: Evaluation, Optimization and Validation of Sample Preparation Methods and Mass Spectrometric Parameters

Himabindu Vasuki Kilambi[#], Kalyani Manda[#], Hemalatha Sanivarapu, Vineet Kumar Maurya, Rameshwar Sharma and Yellamaraju Sreelakshmi*

Repository of Tomato Genomics Resources, Department of Plant Sciences, School of Life Sciences, University of Hyderabad, Hyderabad-500046, India

[#]equal contribution by these authors

***Corresponding author at:** Repository of Tomato Genomics Resources, Department of Plant Sciences, University of Hyderabad, Hyderabad-500046, India. Tel: +91-40-23134771, Fax: +91-40-23010120.

Authors' email address

HVK, h.vasuki2@gmail.com, KM, nidhimanda19@gmail.com, HS, hemalatha.adr@gmail.com, VKM, vineetkm2000@gmail.com, RS rameshwar.sharma@gmail.com, YS, syellamaraju@gmail.com.

Running head: Shotgun Proteomics of Tomato Fruits

A

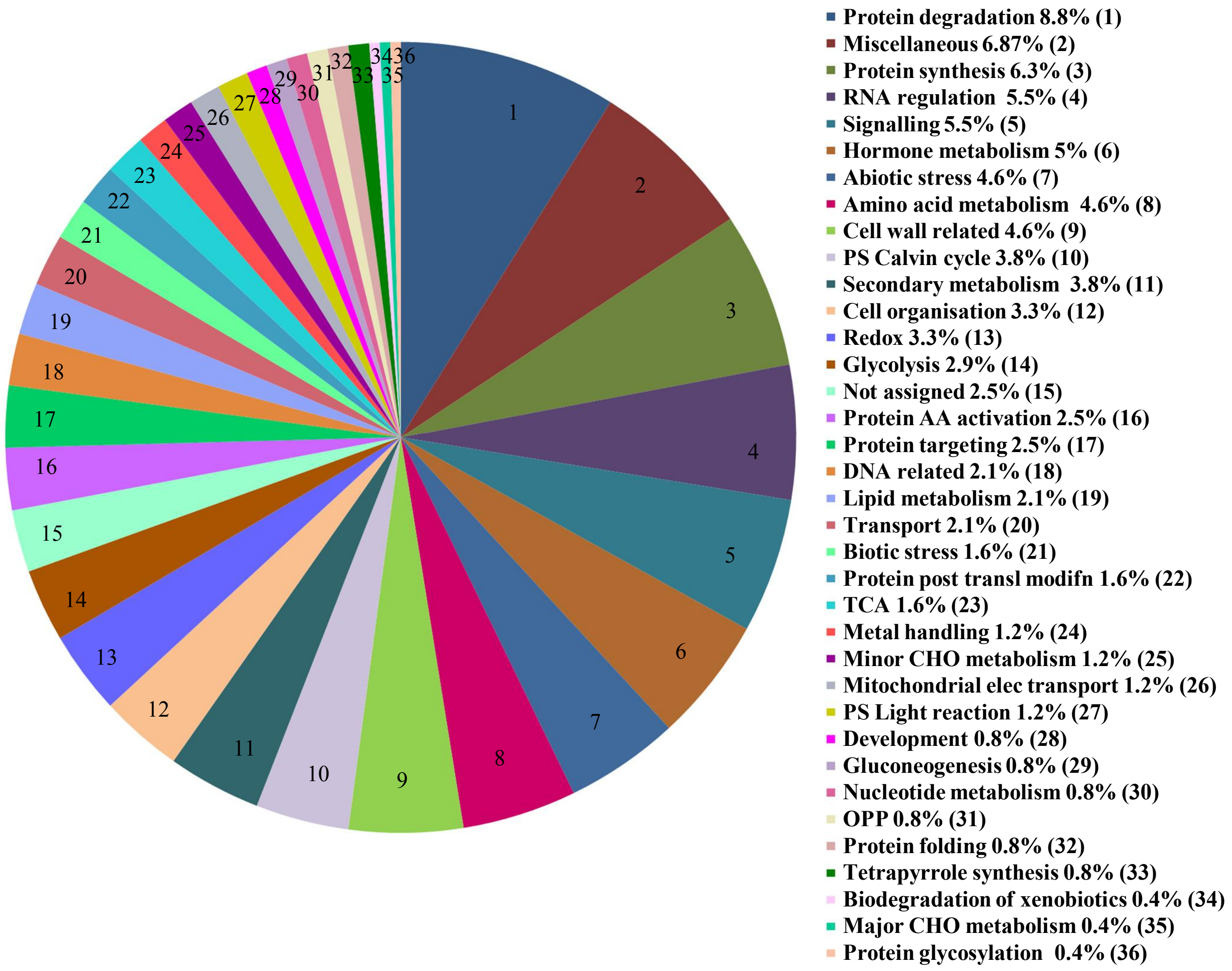

Protein degradation 8.8% (1)
Miscellaneous 6.87% (2)
Protein synthesis 6.3% (3)
RNA regulation 5.5% (4)
Signalling 5.5% (5)
Hormone metabolism 5% (6)
Abiotic stress 4.6% (7)
Amino acid metabolism 4.6% (8)
Cell wall related 4.6% (9)
PS Calvin cycle 3.8% (10)
Secondary metabolism 3.8% (11)
Cell organisation 3.3% (12)
Redox 3.3% (13)
Glycolysis 2.9% (14)
Not assigned 2.5% (15)
Protein AA activation 2.5% (16)
Protein targeting 2.5% (17)
DNA related 2.1% (18)
Lipid metabolism 2.1% (19)
Transport 2.1% (20)
Biotic stress 1.6% (21)
Protein post transl modifn 1.6% (22)
TCA 1.6% (23)
Metal handling 1.2% (24)
Minor CHO metabolism 1.2% (25)
Mitochondrial elec transport 1.2% (26)
PS Light reaction 1.2% (27)
Development 0.8% (28)
Gluconeogenesis 0.8% (29)
Nucleotide metabolism 0.8% (30)
OPP 0.8% (31)
Protein folding 0.8% (32)
Tetrapyrrole synthesis 0.8% (33)
Biodegradation of xenobiotics 0.4% (34)
Major CHO metabolism 0.4% (35)
Protein glycosylation 0.4% (36)

Figure S3.

B

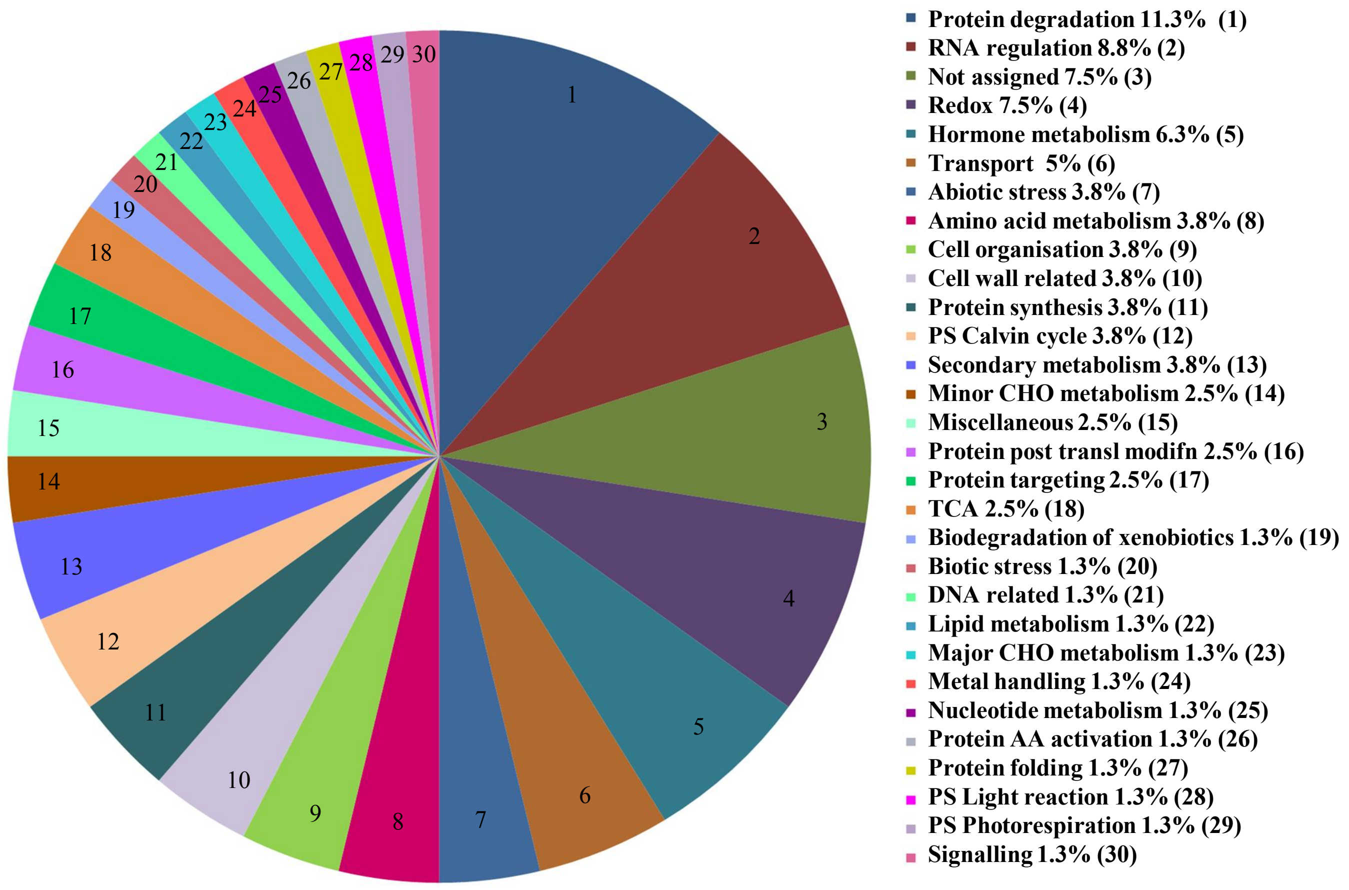

Figure S3. Functional classification of differentially expressed proteins in AV in comparison to AC. **A**. Proteins upregulated in AV in comparison to AC. **B.** Proteins downregulated in AV in comparison with AC
