# Supplementary material for: Shotgun Proteomics of Tomato Fruits: Evaluation, Optimization and Validation of Sample Preparation Methods and Mass Spectrometric Parameters
Source: Front Plant Sci. 2016 Jun 29;7:969. doi: 10.3389/fpls.2016.00969 (PMC4925719; doi:10.3389/fpls.2016.00969)
Supplement: Supplementary file 6 [file Presentation4.PDF]

Shotgun Proteomics of Tomato Fruits: Evaluation, Optimization and Validation of Sample Preparation Methods and Mass Spectrometric Parameters

Himabindu Vasuki Kilambi[#], Kalyani Manda[#], Hemalatha Sanivarapu, Vineet Kumar Maurya, Rameshwar Sharma and Yellamaraju Sreelakshmi*

Repository of Tomato Genomics Resources, Department of Plant Sciences, School of Life Sciences, University of Hyderabad, Hyderabad-500046, India

[#]equal contribution by these authors

***Corresponding author at:** Repository of Tomato Genomics Resources, Department of Plant Sciences, University of Hyderabad, Hyderabad-500046, India. Tel: +91-40-23134771, Fax: +91-40-23010120.

Authors' email address

HVK, h.vasuki2@gmail.com, KM, nidhimanda19@gmail.com, HS, hemalatha.adr@gmail.com, VKM, vineetkm2000@gmail.com, RS rameshwar.sharma@gmail.com, YS, syellamaraju@gmail.com.

Running head: Shotgun Proteomics of Tomato Fruits

A

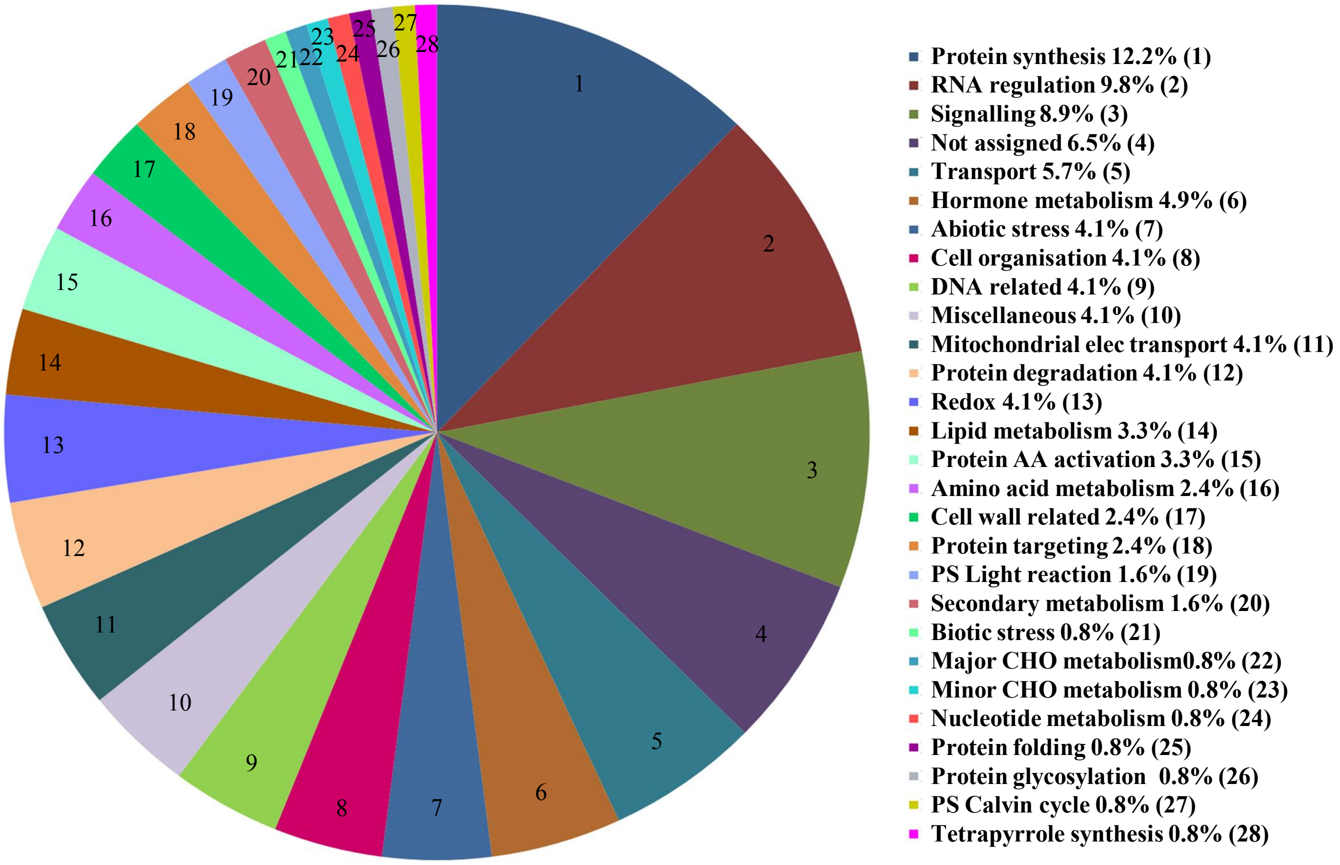

1
2
3
4
5
6
7
8
9
10
11
12
13
14
15
16
17
18
19
20
21
22
23
24
25
26
27
28
Protein synthesis 12.2% (1)
RNA regulation 9.8% (2)
Signalling 8.9% (3)
Not assigned 6.5% (4)
Transport 5.7% (5)
Hormone metabolism 4.9% (6)
Abiotic stress 4.1% (7)
Cell organisation 4.1% (8)
DNA related 4.1% (9)
Miscellaneous 4.1% (10)
Mitochondrial elec transport 4.1% (11)
Protein degradation 4.1% (12)
Redox 4.1% (13)
Lipid metabolism 3.3% (14)
Protein AA activation 3.3% (15)
Amino acid metabolism 2.4% (16)
Cell wall related 2.4% (17)
Protein targeting 2.4% (18)
PS Light reaction 1.6% (19)
Secondary metabolism 1.6% (20)
Biotic stress 0.8% (21)
Major CHO metabolism0.8% (22)
Minor CHO metabolism 0.8% (23)
Nucleotide metabolism 0.8% (24)
Protein folding 0.8% (25)
Protein glycosylation 0.8% (26)
PS Calvin cycle 0.8% (27)
Tetrapyrrole synthesis 0.8% (28)

Figure S4.

B

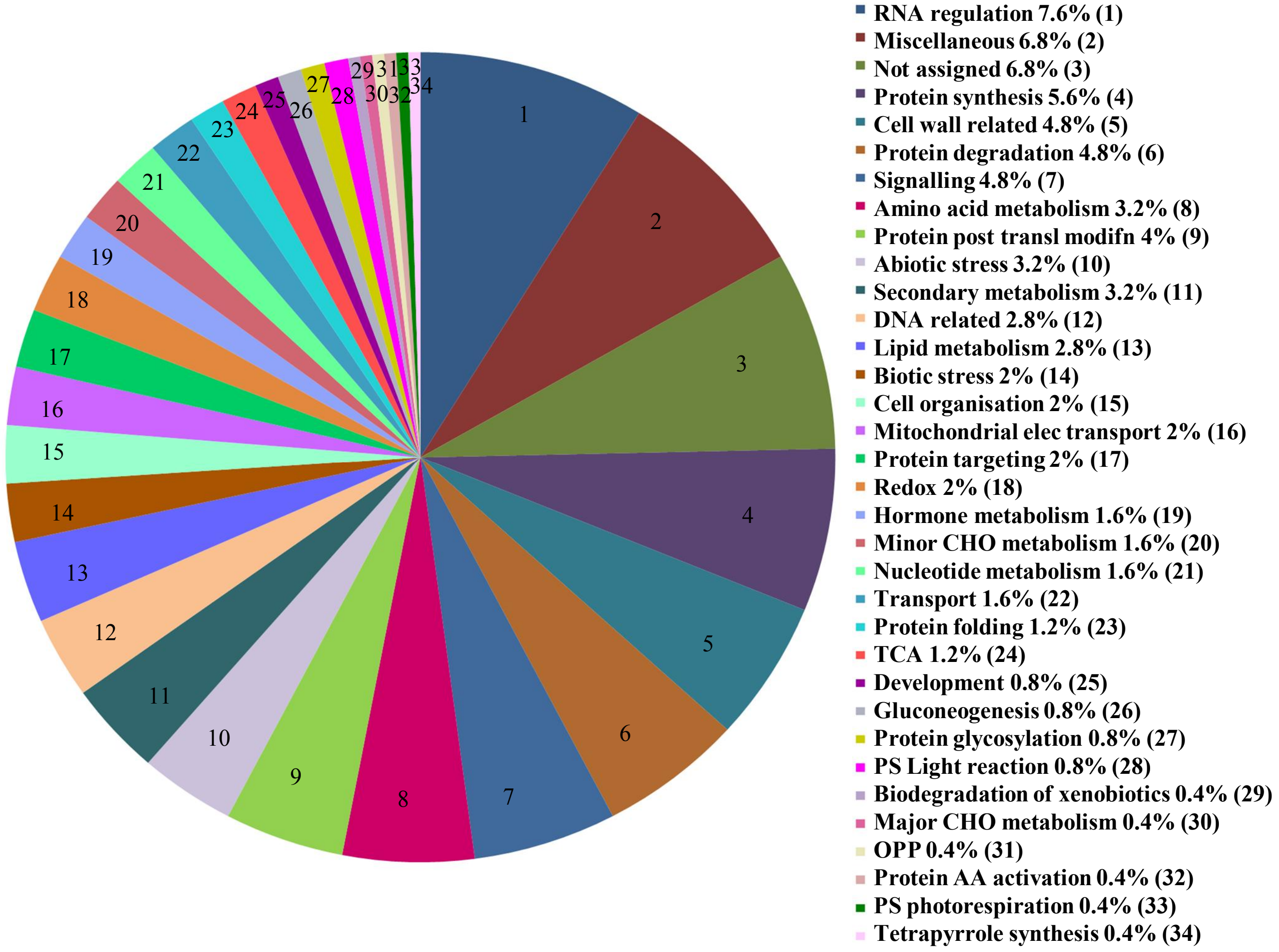

Figure S4 Functional classification of proteins identified in only one cultivar. **A**. Proteins identified only in AC. **B.** Proteins identified only in AV
